# NAST 2.0: Magnetic Field Effects and Marcus–Levich–Jortner Theory for Complex Open-Shell Systems

**DOI:** 10.1021/acsomega.6c07270

**Published:** 2026-09-20

**Authors:** Ilya D. Dergachev, Vsevolod D. Dergachev, Mitra Rooein, Yash H. Patel, Philip S. Weiss, Claudia E. Avalos, Sergey A. Varganov

**Affiliations:** † Molecular Design Institute, Department of Chemistry, 5894 New York University, New York City 10003, New York, United States; ‡ Department of Chemistry, University of Nevada, Reno, Reno 89557-0216, Nevada, United States; § Department of Chemistry for Life Sciences, 8097 Uppsala University, 75123 Uppsala, Sweden

## Abstract

We present a new version of the nast software package for predicting the kinetics of unimolecular spin-dependent processes, including intersystem crossings, spin-forbidden reactions, and spin crossovers with nonadiabatic statistical theory. With substantial extensions and upgrades, the new nast 2.0 release enables the calculations of (1) rate constants for spin-dependent reactions in an external magnetic field, (2) Marcus–Levich–Jortner rate constants with a new mlj program for complex open-shell systems, (3) Huang–Rhys factors and reorganization energies with a new *vib* tool, (4) Wigner and Eckart quantum tunneling correction coefficients, (5) minimum energy crossing points with a new pymecp program interfaced with the orca electronic structure package, (6) effective Hessian with the q-chem and orca packages, (7) intrinsic reaction coordinate fits with the orca package for more advanced nast calculations with the Zhu–Nakamura transition probabilities, and (8) transition probabilities and rate constants with integration step-size control. Together, these capabilities significantly enhance the applicability of nast for studying the kinetics of spin-dependent processes even in complex open-shell systems. This paper describes the objectives and applications of the new capabilities illustrated with numerical examples. nast is an open-source software package and is available free of charge.

## Graphical abstract

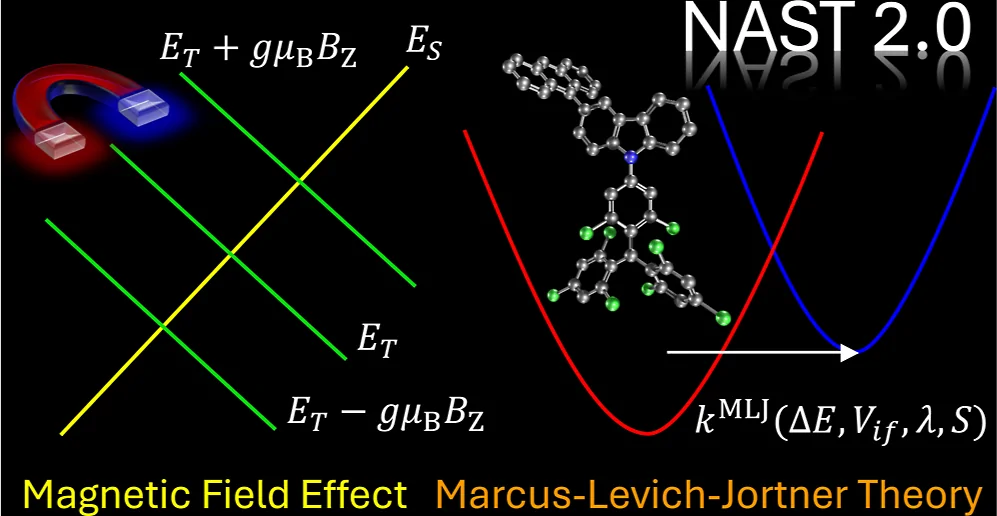

## 1 Introduction

The nonadiabatic statistical theory (NAST) is an inexpensive yet powerful tool for predicting kinetics of unimolecular spin-dependent processes such as intersystem crossing transitions, spin-forbidden reactions, and spin crossovers.^1–9^ Similar to transition state theory (TST), NAST requires the molecular properties at only a few points along the reaction coordinate (Figure 1). In the simplest case, NAST requires only the Hessians at the reactant minimum and the minimum energy crossing point (MECP) geometries and the spin–orbit coupling constant at the MECP. This makes NAST well-suited for very large systems consisting of hundreds and thousands of atoms, such as solvated proteins.^10^

**Figure 1:**
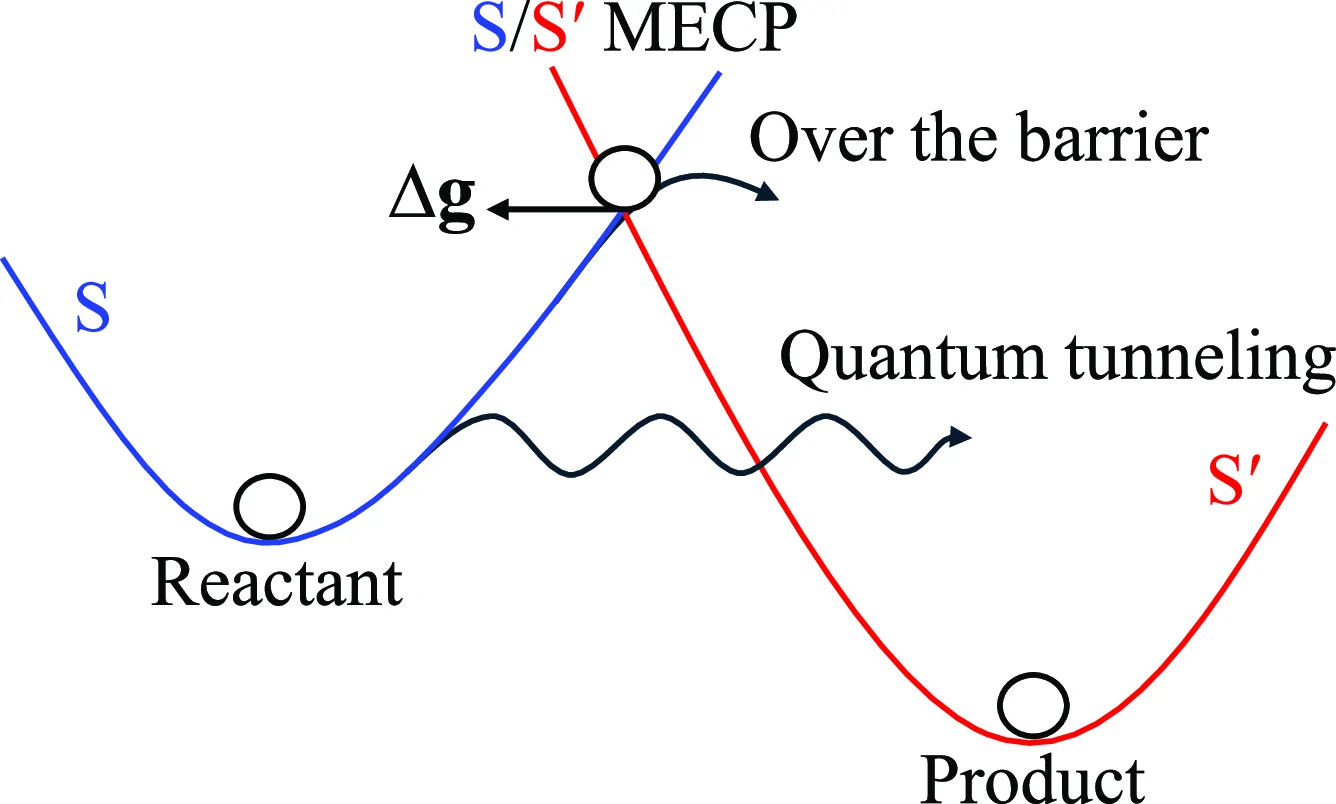
Hypothetical peaked-type intersection of potential energy surfaces of two different spin states S and S’. The reactant and product belong to the surfaces of different spin states but are coupled at the minimum energy crossing point (MECP) due to the spin–orbit interaction. Vector Δ**g** = **g**_1_ – **g**_2_, calculated from the state gradients **g**_1_ and **g**_2_, is the gradient vector parallel to the reaction coordinate at the MECP. The bent arrow shows the classical over-the-barrier reaction pathway, and the wavy curve shows the tunneling pathway through the MECP energy barrier.

Like TST, NAST assumes that the transition occurs along the reaction coordinate, which connects the reactant’s minimum-energy geometry on the electronic potential energy surface (PES) to the geometry at the energy barrier. In the case of NAST, the energy barrier is formed by the minimum energy crossing point (MECP) between the PESs of two electronic states with different spins (Figure 1). Unlike TST, NAST requires the calculation of the probability of a nonadiabatic transition between the two PESs at the MECP geometry. The two most commonly employed methods for calculating this probability are the Landau–Zener (*P*_LZ_) and weak coupling (*P*_WC_) formulas. In the so-called “double-passage” form, named as such because it accounts for the probability of transitions at the MECP in both the forward and reverse directions, these formulas are expressed as follows:^11–13^

1 $$P_{LZ}\left(\epsilon_{⊥}\right)=1-\exp\left(-\frac{4\pi H_{\text{SO}}^{2}}{\hbar \left|\Delta g\right|}\sqrt{\frac{\mu_{⊥}}{2\left(\epsilon_{⊥}-E_{X}\right)}}\right)$$

$$P_{WC}\left(\epsilon_{⊥}\right)=4\pi^{2}H_{\text{SO}}^{2}{\left(\frac{2\mu_{⊥}}{\hbar^{2}\mathrm{g̅}\left|\Delta g\right|}\right)}^{2/3}\times {\left[Ai\left(-\left(\epsilon_{⊥}-E_{X}\right){\left(\frac{2\mu_{⊥}{\left|\Delta g\right|}^{2}}{\hbar^{2}{\mathrm{g̅}}^{4}}\right)}^{1/3}\right)\right]}^{2}$$ 2

where *H*_SO_ is the spin–orbit coupling constant, ℏ is the reduced Planck constant, $\left|\Delta g\right|=\left|g_{1}-g_{2}\right|$ is the norm of the gradient parallel to the reaction coordinate at the MECP, $\mathrm{g̅}={\left(|g_{1}∥g_{2}|\right)}^{1/2}$ is the geometric mean of the two spin state gradients **g**_1_ and **g**_2_, *μ*_⊥_ is the reduced mass for the reaction coordinate, *ϵ*_⊥_ and *E*_X_ are the energy along the reaction coordinate and the MECP energy barrier, respectively, and Ai is the Airy function of the first kind.

The microcanonical (energy-dependent) *k*(E) and canonical (temperature-dependent) *k*(T) rate constants are calculated as follows:

3 $$k\left(E\right)=\sigma \frac{N_{X}\left(E\right)}{h\rho_{R}\left(E\right)}$$

4 $$k\left(T\right)=\frac{\sigma }{hQ_{R}\left(T\right)}\int_{0}^{\infty }N_{X}\left(E\right)e^{-E/k_{B}T}dE$$

5 $$N_{X}\left(E\right)=\int_{0}^{E}\rho_{X}\left(E-\epsilon_{⊥}\right)P\left(\epsilon_{⊥}\right)d\epsilon_{⊥}$$

where *E* is the internal (rovibrational) energy, *N*_X_(*E*) is the effective number of states at the MECP, *ρ*_R_ and *ρ*_X_ are the densities of rovibrational states for the reactant and MECP, respectively, *h* is the Planck’s constant, *Q*_R_ is the partition function of the reactant, *k*_B_ is the Boltzmann constant, *T* is the temperature, and *σ* is the reaction path symmetry number.^7^

Both the *P*_LZ_ and *P*_WC_ formulas have certain assumptions and limitations which limit their applicability. *P*_LZ_ is non-zero only at *ϵ*_⊥_ above the MECP energy, i.e., only at energies greater than the energy barrier, and therefore, *k*_LZ_ is a semi-classical “over-the-barrier rate constant” which importantly does *not* account for quantum tunneling through the MECP barrier. As a result, the most accurate *k*_LZ_ values are expected for systems where the role of quantum tunneling is insignificant. Furthermore, *P*_LZ_ lacks the effect of quantum interference between primary and secondary passages at the MECP.^7^ *P*_WC_, in contrast, accounts for both quantum effects but is limited in application to only relatively weak spin–orbit coupling due to the quadratic dependence of *P*_WC_ on *H*_SO_ (eq 2). Both the forms of *P*_LZ_ and *P*_WC_ assume a linear shape of crossing potentials at all energies. For this reason, *P*_WC_ can be potentially overestimated at lower temperatures due to the increased quantum tunneling through the artificially narrow barrier. Thankfully, many limitations of both the *P*_LZ_ and *P*_WC_ formulas can be addressed with the use of the advanced Zhu–Nakamura (ZN) formulas for nonadiabatic transition probabilities.^14–19^ However, application of the ZN formulas requires an explicit reaction potential and differs for peaked-type and sloped-type intersections. Currently, only sloped-type calculations with the ZN formulas are available in nast. The detailed description of nonadiabatic statistical theory and the workflow of the nast package are given in our first release paper.^7^ A brief description of the nast package structure is given in the Supporting Information. In the rest of the paper, we describe the new capabilities added to nast since its first release. These include the following:

1. Spin-dependent rate constants under external magnetic fields;
2. The mlj program for calculating Marcus–Levich–Jortner rate constants;
3. The *vib* tool for calculating the Huang–Rhys factors and reorganization energies;
4. Wigner and Eckart quantum tunneling corrections to transition state theory rate constants;
5. The pymecp program for searching the MECP geometry with the orca electronic structure package;
6. The effective Hessian tool *effhess* is extended to work with q-chem and orca electronic structure packages;
7. The intrinsic reaction coordinate tool *irc* is extended to work with orca;
8. Integration step size control.

## 2 New Capabilities

### 2.1 Magnetic Field Effect

Recently, we introduced a theoretical framework to describe the effects of an applied magnetic field on the kinetics of spin-dependent processes using NAST.^4^ The new framework accounts for both spin–orbit and Zeeman couplings between the spin-diabatic states for the singlet–triplet transitions. The total electronic Hamiltonian reads:

6 $$H_{0}+H_{SO}+H_{Z}=\left(\begin{array}{llll} E_{S} & z & ib & z^{*}\\ z^{*} & E_{T}-g\mu_{B}B_{z} & \frac{g\mu_{B}B_{+}}{\sqrt{2}} & 0\\ -ib & \frac{g\mu_{B}B_{-}}{\sqrt{2}} & E_{T} & \frac{g\mu_{B}B_{+}}{\sqrt{2}}\\ z & 0 & \frac{g\mu_{B}B_{-}}{\sqrt{2}} & E_{T}+g\mu_{B}B_{z} \end{array}\right)$$

where *E*_*S*_ and *E*_*T*_ are the energies of the singlet |*S*⟩ and three components of the triplet states |*T*_0,±1_⟩, respectively, *z* and *ib* are the spin–orbit coupling matrix elements,^6^ *g* is the free electron g-factor, *μ*_*B*_ is the Bohr magneton, *B*_*Z*_ is the strength of the applied magnetic field along the *z*-axis, and *B*_±_ = *B*_*x*_ ± *iB*_*y*_. Diagonalization of the Hamiltonian matrix in eq 6 yields four adiabatic electronic states with energies *E*_1_, *E*_2_, *E*_3_, and *E*_4_, which depend on both the applied magnetic field and the spin–orbit coupling matrix elements. We introduce two dimensionless parameters, *m* and *n*, which characterize the strength of the applied magnetic field relative to the spin–orbit coupling and the ratio of spin–orbit coupling matrix elements between the singlet state and different *M*_*S*_ components of the triplet state:^4^

7 $$m=\frac{g\mu_{B}B_{Z}}{SOC_{eff}}$$

8 $$\text{SO}C_{eff}=\sqrt{2\left|z{|}^{2}+\right|b{|}^{2}}$$

9 $$n=\frac{\left|z\right|}{\left|b\right|}$$

The combination of *m* and *n* defines six distinct coupling regimes, as illustrated in Figure 2. When analyzing the magnetic field effect on the kinetics of a spin-dependent unimolecular reaction (assuming *B*_*z*_ ≠ 0 and *B*_*x*,*y*_ = 0), three magnetic field regimes, weak, intermediate, and strong, can be distinguished on the basis of the value of *m* (eq 7). Each case exhibits different energy profiles for the four adiabatic states, and three different approaches can be employed to calculate transition probabilities and rate constants. The values of *m* that separate the three regimes are system-specific.

**Figure 2:**
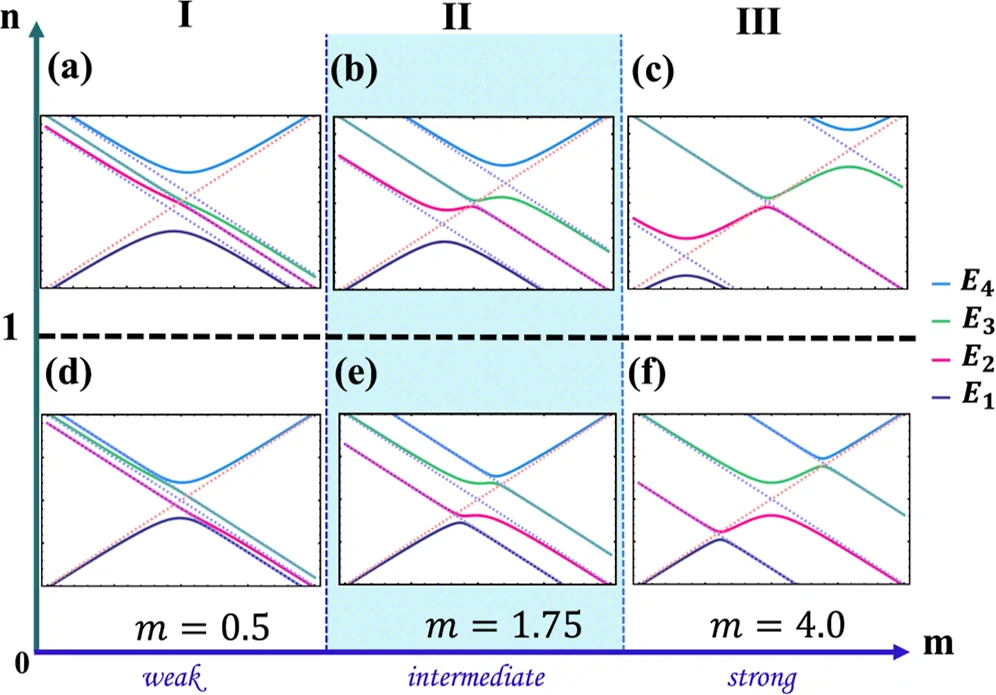
Six coupling regimes (a)–(f) based on the values of the relative magnetic field strength *m* and the relative spin–orbit coupling strengths between *M*_*S*_ components of singlet and triplet states *n*. Weak, intermediate, and strong magnetic field regimes can be defined by the value of *m*. Solid lines show adiabatic energies for four states as a function of a reaction coordinate, and dotted lines show Zeeman spin-diabatic energies. Adapted with permission from ref 4. Copyright 2024 AIP Publishing.

Theoretical approaches for predicting transition probabilities and rate constants in the weak and strong magnetic field regimes are described in detail in our recent work.^4^ As an example, it was shown that the application of a magnetic field to the spin-forbidden isomerization of the Ni­(dpp)­Cl_2_ complex [dpp = 1,3-bis­(diphenylphosphino)­propane] is predicted to increase the rate constant by 10% at 50 T. The new framework, implemented in the present release of the nast package, provides a systematic methodology for predicting the kinetics of spin-dependent reactions in external magnetic fields.

### 2.2 The mlj Program

The development of a new program called mlj for calculating rate constants within the Marcus–Levich–Jortner (MLJ) theory was motivated by extensive studies of photoexcited chromophore–radical systems. Such systems play an important role in advancing optoelectronic and molecular spintronic technologies, with promising applications in quantum sensing, coherent spin control, and magnetic resonance signal enhancement.^20–26^ However, locating minimum energy crossing point (MECP) geometries in chromophore–radical systems can be particularly challenging due to their open-shell electronic structure, which often leads to spin contamination and pronounced multi-configurational character. In addition, the small energy gaps and strong electronic similarity between the interacting states make the crossing region shallow and numerically difficult to resolve with computationally feasible single-reference methods like time-dependent density functional theory.

The MLJ theory roots in the Fermi’s Golden Rule theory and does not require the knowledge of the MECP. The MLJ theory is widely used for modeling nonadiabatic transitions in molecular systems, particularly where both classical and quantum nuclear motions influence reaction dynamics.^27–32^ It improves upon classical Marcus theory via explicit quantum-mechanical treatment of the high-frequency intramolecular vibrational modes, while still treating low-frequency environmental modes classically. The MLJ theory has proven valuable in revealing mechanisms of photoinduced processes, including luminescence and spin polarization mechanisms in neutral organic radicals.^33–36^ The MLJ rate constant is calculated as:

$$k_{MLJ}\left(T\right)=\frac{2\pi }{\hbar }{\left|V_{if}\right|}^{2}\frac{1}{\sqrt{4\pi \lambda k_{B}T}}\times \overset{\infty }{\underset{n=0}{\sum }}\frac{e^{-S_{eff}}S_{eff}^{n}}{n!}\exp\left[-\frac{{\left(\Delta E+\lambda +n\hbar \omega_{eff}\right)}^{2}}{4\lambda k_{B}T}\right]$$ 10

where ℏ is the reduced Planck constant; *V*_*if*_ ≡ $\left\langle \phi_{i}\left|\mathrm{V̂}\right|\phi_{f}\right\rangle$ is the matrix element of the operator V̂, which couples initial and final electronic states *ϕ*_*i*_ and *ϕ*_*f*_; λ is the classical reorganization energy; and *n*, *S*_eff_, and ω_eff_ are the vibrational quantum number, Huang–Rhys factor, and frequency of the single effective high-frequency vibrational mode, respectively. It is important to note that the MLJ theory, as well as Marcus theory, can be viewed as special case of a more general full-dimensional harmonic approximation of FGR introduced by Marian and coworkers.^37^

We note that the MLJ rate constant in eq 10 is evaluated in its low-temperature, emission-only form, in which the vibronic sum runs over *n* ≥ 0 with Poisson weights *e* ^–*S*^ *S* ^*n*^/*n*! describing an effective mode that begins in its vibrational ground state and gains *n* quanta of energy ℏω during the transition.^29^ This form is most accurate for exergonic steps (Δ*E* < 0): the resonant channel then lies at positive *n*, so the released electronic energy is emitted into the mode and is captured completely by the *n* ≥ 0 series. For the corresponding endergonic (reverse) step, the resonant channel would instead require *n* < 0 absorption of quanta from a thermally excited mode, which is absent from the emission-only series. Evaluating the same expression at +Δ*E* retains only the classical (*n* = 0) activation term and therefore systematically underestimates the reverse rate, increasingly so as the temperature is lowered. The reverse rate can be obtained from the forward rate via detailed balance, *k*_rev_ = *k*_fwd_exp­(Δ*E*/*k*_B_ *T*), where Δ*E* < 0 (the driving force for the forward process), thereby ensuring thermal equilibrium between the two electronic states.^38–41^

The mlj program is a separate program distributed with the NAST package and is described in detail in the nast manual. The mlj program requires only the diabatic electronic energies of the two electronic states of different total spins, vibrational frequencies, and electronic couplings as input from electronic structure calculations. To make nast as streamlined and self-contained a tool as possible, Huang–Rhys factors and reorganization energies can now be obtained from our new tool, *vib*, which is also distributed with nast and is described in the section below.

In this section, we demonstrate the application of the mlj program with the example of a spin–optical interface based on the tris­(2,4,6-trichlorophenyl)­methyl–carbazole (TTM-1Cz) luminescent organic radical coupled to an anthracene (An) chromophore (Figure 3a). Upon photoexcitation, the TTM-1Cz–An system is promoted from its ground ^2^[*D*_0_ *S*_0_] state to an emissive doublet excited state, ^2^[*D*_1_ *S*_0_], characterized by a local *D*_1_ excitation on the TTM-1Cz moiety, while the anthracene chromophore remains in its closed-shell *S*_0_ state as shown in Figure 3b,c. Population transfer from the photoexcited ^2^[*D*_1_ *S*_0_] state to the ^2^[*D*_0_ *T*_1_] state, which features a local open-shell *T*_1_ excitation on the anthracene unit, occurs on a time scale of approximately one picosecond, as evidenced by transient absorption spectroscopy.^34^ Subsequently, intersystem crossing drives the transition from the ^2^[*D*_0_ *T*_1_] state (*S*_TOT_ = 1/2) to the quartet ^4^[*D*_0_ *T*_1_] state (*S*_TOT_ = 3/2).^42^ This process may be influenced by the energetic proximity of the charge-transfer (CT) state ^2^ *CT*. The resulting high-spin ^4^[*D*_0_ *T*_1_] state can be coherently manipulated and read out optically, as demonstrated in the recent work.^34^ Owing to the close energetic spacing of the four states shown in Figure 3c, the emissive ^2^[*D*_1_ *S*_0_] state can be repopulated; this reverse internal conversion step enables optical readout of the spin states of the ^4^[*D*_0_ *T*_1_] manifold. Thus, the TTM-1Cz–An open-shell system constitutes an example of a spin-optical interface realized through the coupling of a stable luminescent organic radical with an organic chromophore.

**Figure 3:**
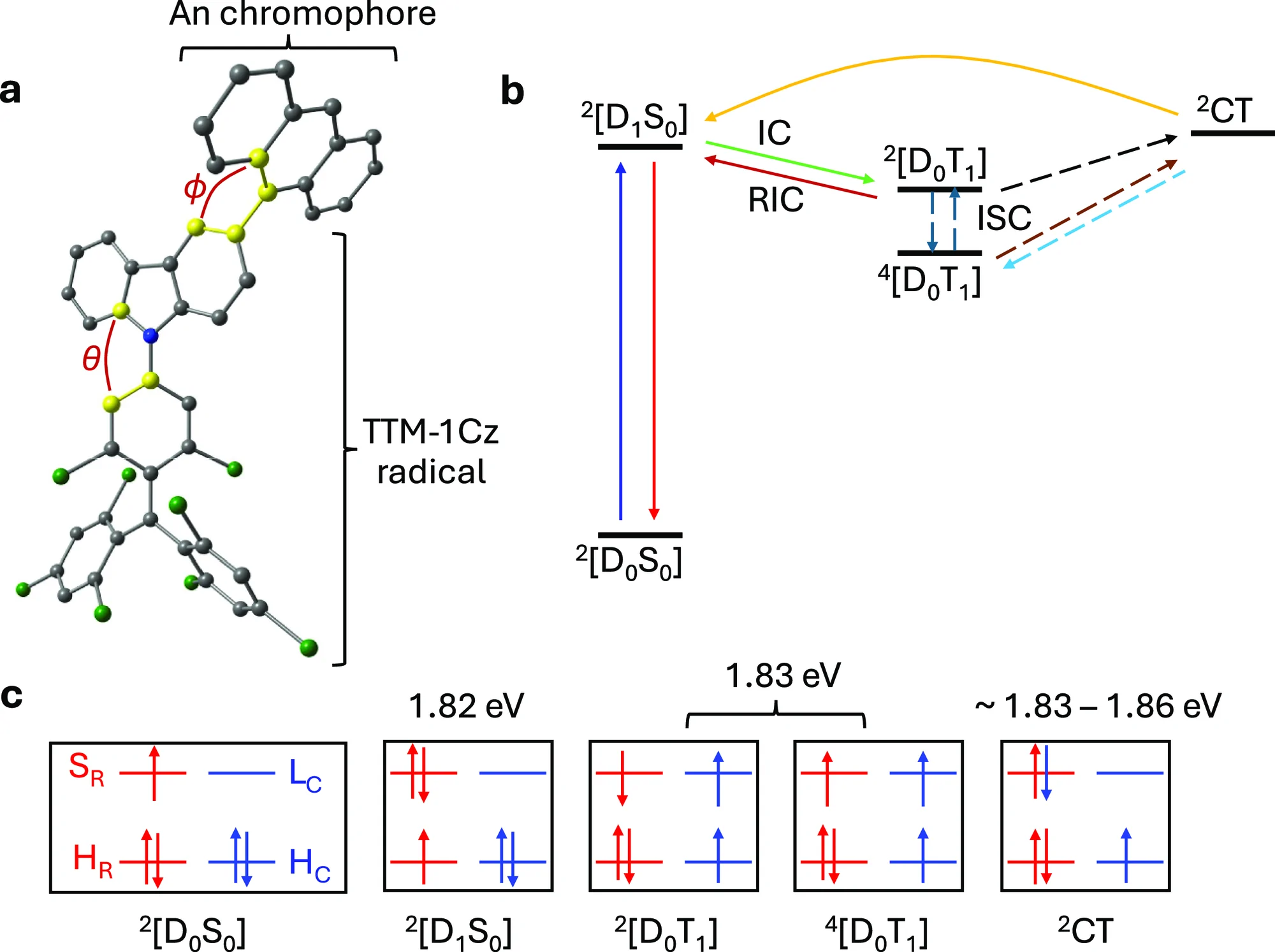
(a) The TDA-optimized geometry of the excited radical doublet state ^2^[*D*_1_ *S*_0_] of the TTM-1Cz-An chromophore-radical system. Gray, blue, and green atoms indicate carbon, nitrogen, and chlorine atoms, respectively. The hydrogen atoms are omitted for clarity. The carbon atoms in yellow and also the nitrogen atom define the dihedral angles θ and ϕ between the TTM and 1-Cz moieties and the 1-Cz and An moieties, respectively. (b) The energy level diagram of the states involved in the spin-dependent photoluminescence (PL) mechanism of the TTM-1Cz-An radical. Reverse internal conversion (RIC) is one of the mechanisms of the repopulation of the emissive ^2^[*D*_1_ *S*_0_] state due to the close proximity of the ^2^[*D*_0_ *T*_1_] state, as well as the charge transfer (CT) from the ^2^ *CT* state. (c) Principal electronic configurations of the states involved in the PL mechanism. H_R_ and S_R_ are the highest occupied and singly occupied molecular orbitals of the radical, and H_L_ and L_C_ are the highest occupied and the lowest unoccupied molecular orbitals of the chromophore, respectively.

In this example, we test the mlj program by calculating the rate constant for the ^2^[*D*_0_ *T*_1_] → ^2^[*D*_1_ *S*_0_] reverse internal conversion (RIC) transition in the TTM-1Cz-An radical and comparing it to the previously reported values.^34^ The close energetic proximity and electronic coupling of these two states can lead to the repopulation of the emissive ^2^[*D*_1_ *S*_0_] state by the RIC from the ^2^[*D*_0_ *T*_1_] state and contribute to the efficient luminescence of the TTM-1Cz-An radical, enabling its optical readout with optically detected magnetic resonance (ODMR) spectroscopy.

Calculation of Huang–Rhys factors, vibrational reorganization energies, and the energy gap for the ^2^[*D*_0_ *T*_1_] → ^2^[*D*_1_ *S*_0_] RIC requires relaxed geometries corresponding to the energy minima of the ^2^[*D*_0_ *T*_1_] and ^2^[*D*_1_ *S*_0_] PES. The optimized geometry of the ^2^[*D*_1_ *S*_0_] state was obtained with time-dependent density functional theory (TD-DFT) under the Tamm–Dancoff approximation (TDA) at the CAM-B3LYP/def2-SVP level of theory. The solvent environment (toluene) was modeled using the conductor-like polarizable continuum model (CPCM) solvation method. The equilibrium geometry of the ^2^[*D*_0_ *T*_1_] state was approximated with the equilibrium geometry of the ^4^[*D*_0_ *T*_1_] state optimized with ground-state DFT (see explanation below). Electronic energies used in the MLJ calculations were obtained from the complete active space self-consistent field method (CASSCF) with an active space made of nine electrons in seven orbitals, coupled with the *N*-electron valence second-order perturbation theory (NEVPT2) and using the def2-TZVP basis set, as previously applied to this system.^34^ The details of the multireference calculations are given in the Supporting Information. All calculations were performed with the ORCA 6.1 electronic structure package.^43–50^ The ORCA input files and the XYZ coordinates of the equilibrium geometries are given in the Supporting Information.

The optimized geometry of the excited ^2^[*D*_0_ *T*_1_] state could not be obtained with TD-DFT since this state contains a configuration with double-excitation character arising from the *S*_0_ → *T*_1_ transition on the chromophore accompanied by the spin-flip of the radical’s electron, which is poorly described by the conventional linear response TD-DFT. In principle, the optimized geometry of the ^2^[*D*_0_ *T*_1_] state could be obtained from the spin-flip TD-DFT (SF-TD-DFT) from the reference ^4^[*D*_0_ *T*_1_] state, which is the lowest quartet spin state. However, this geometry may not be accurate enough due to potential spin contamination common in SF-TD-DFT. On the other hand, weak exchange interactions between the unpaired electrons of the excited chromophore and the lone electron of the radical make the excited trip-doublet and trip-quartet states nearly isoenergetic (Figure 3c), with a typical energy separation ranging from a fraction of meV to a few meV,^26,42^ allowing us to suggest similar minima for the two states. Indeed, the NEVPT2-predicted $\delta E=E\left({}^{2}\left[D_{0}T_{1}\right]\right)-E\left({}^{4}\left[D_{0}T_{1}\right]\right)$ energy difference, calculated at three geometries corresponding to the minima of the ^2^[*D*_0_ *S*_0_], ^2^[*D*_1_ *S*_0_], and ^4^[*D*_0_ *T*_1_] states, did not exceed 1 meV. Thus, the geometry of the ^2^[*D*_0_ *T*_1_] state was approximated with that of the ^4^[*D*_0_ *T*_1_] state with an estimated error of 1 meV.

The conformation of the TTM-1Cz-An radical is characterized in part by two dihedral angles, the angle between the TTM and 1Cz moieties *θ*, and the angle between the 1Cz and An moieties ϕ (Figure 3). The ^2^[*D*_1_ *S*_0_] and ^2^[*D*_0_ *T*_1_] equilibrium geometries exhibited angles θ and ϕ of θ/ϕ = 44°/83° and 53°/89°, respectively. The fully relaxed geometry of the ^2^[*D*_0_ *T*_1_] state exhibited a small imaginary frequency of 8.8*i* cm^–1^ for the vibrational mode along the angle ϕ. However, it has been shown that the energy of the ^2^[*D*_0_ *T*_1_] state stays nearly constant for the angle ϕ ranging from 60° to 90°.^34^ Also, in our case, the small difference between the equilibrium geometries of ^2^[*D*_1_ *S*_0_] and ^2^[*D*_0_ *T*_1_] states in terms of the angle ϕ (83° vs. 89°) minimizes the reorganization energy between the two states. The energy gap between two states was found to be 28 meV, slightly less than the predicted 60 meV gap estimated for the closest pair of geometries reported in the previous study with θ/ϕ = 40°/78° for ^2^[*D*_1_ *S*_0_] and 52°/90° for ^2^[*D*_0_ *T*_1_].^34^ Huang–Rhys factors and reorganization energy were computed using the *vib* tool distributed with NAST and described in the section below. We used the electronic coupling constant of 5 meV previously reported for the θ/ϕ = 52°/90° geometry.^34^ The MLJ rate constant of 2.7 × 10^9^ s^–1^ was predicted in good agreement with the constant of 6.6 × 10^9^ s^–1^ reported in the previous work for the θ/ϕ = 52°/90° geometry.^34^ The absolute relative error |*k*(Δ*E*+δ*E*)-*k*(Δ*E*))/*k*(Δ*E*)| in the MLJ rate constant associated with approximating the equilibrium geometry of the ^2^[*D*_0_ *T*_1_] state with that of the ^4^[*D*_0_ *T*_1_] state was estimated as 1.4% for Δ*E* = 28 meV, δ*E* = 1 meV, λ = 204 meV, and *n* = 10 used in this work.

### 2.3 Tool *vib*

The *vib* program is a new tool distributed with the NAST package in addition to the *effhess* and *ircfit* tools introduced in the first release. The *vib* tool is designed to calculate the Huang–Rhys factors *S*_*k*_ and reorganization energies λ_*k*_ for 3N-6 (or in the case of a linear molecule 3N-5) vibrational modes *Q*_*k*_ of a molecule:

11 $$S_{k}=\frac{1}{2}\frac{\omega_{k}}{\hbar }{\left(\Delta Q_{k}\right)}^{2}$$

12 $$\lambda_{k}=\frac{1}{2}\omega_{k}^{2}{\left(\Delta Q_{k}\right)}^{2}$$

where ω_*k*_ is the vibrational frequency of the *k*th normal mode, and Δ*Q*_*k*_ is the distance between minima of two electronic states along this mode. Huang–Rhys factors and reorganization energies calculated with *vib* are then used for calculating rate constants with the mlj program. To calculate *S*_*k*_ and λ_*k*_, *vib* only requires the XYZ coordinates of the optimized geometries for two spin states and the single Hessian matrix for one of the states, under the assumption that both electronic spin states are described by the same harmonic oscillator. The Huang–Rhys factors can be visualized for a convenient analysis of which vibrational modes lead to the largest nuclear overlap of two electronic states, as shown in Figure 4 in the example of the ^2^[*D*_0_ *T*_1_] → ^2^[*D*_1_ *S*_0_] transition in the TTM-1Cz-An molecule.

**Figure 4:**
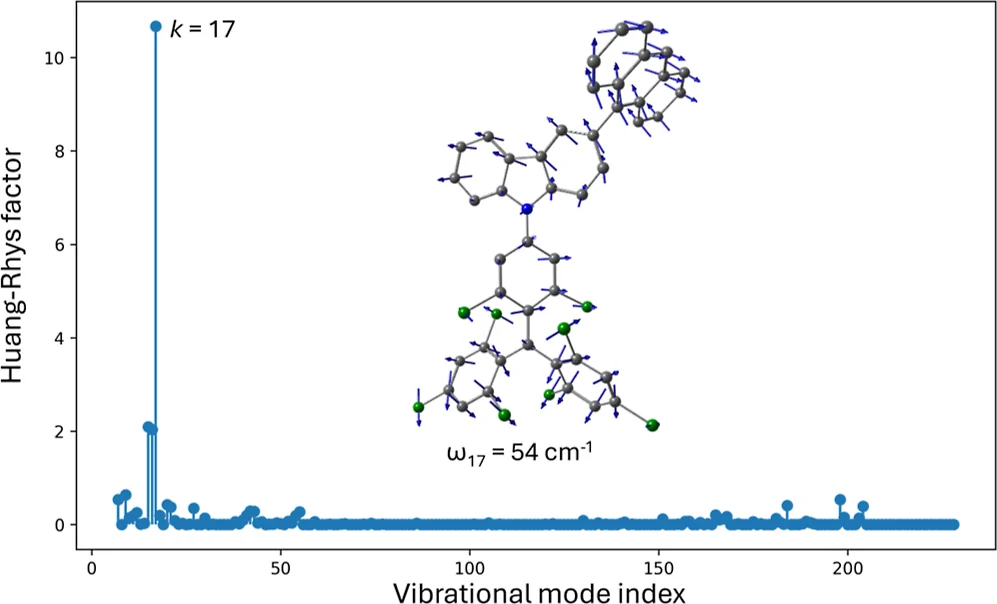
Huang–Rhys factors for the vibrational overlap between the relaxed geometries of the ^2^[*D*_0_ *T*_1_] and ^2^[*D*_1_ *S*_0_] states in the TTM-1Cz-An molecule. The largest Huang–Rhys factor is predicted for the vibrational mode Q_17_. Blue arrows show the displacements of atoms along this normal mode of vibration.

Figure 4 shows that the largest overlap between the reactant (^2^[*D*_0_ *T*_1_]) and product (^2^[*D*_1_ *S*_0_]) geometries, quantified by the largest Huang–Rhys factor *S*, occurs along the low-frequency vibrational mode Q_17_. This mode can be described as the simultaneous twisting of the TTM moiety and bending of both 1Cz and An moieties. Vibrational frequencies, Huang–Rhys factors, and reorganization energies for all TTM-1Cz-An modes are given in the Supporting Information. When the reactant and product geometries differ only by small structural relaxations and no bond breaking or formation occurs, their vibrational normal modes remain nearly identical and can be described using a single Hessian. In this case, a Huang–Rhys factor can be computed for each of the vibrational modes by projecting the geometry displacement along this mode according to eq 11. For larger structural changes, the vibrational coordinates of the two states are no longer equivalent, and an explicit treatment of Duschinsky rotation is required to account for the normal mode mixing:^51,52^

13 $$Q_{f}=JQ_{i}+D$$

where ***Q***_*i*_ and ***Q***_*f*_ are the normal coordinates of the initial and final states, respectively, **J** is the Duschinsky rotation matrix, and ***D*** is the displacement vector. Duschinsky transformation may be performed according to the procedures outlined in literature^53–55^ as well as with the software packages such as DUSHIN^54^ or MOMAP.^55^

### 2.4 Quantum Tunneling Corrections

Quantum tunneling (QT) refers to the existence of a nonzero amplitude of a wavefunction across a potential energy barrier at energies below the barrier height. In the context of chemical dynamics, this results in population transfer from a reactant to a product state at energies below the activation energy. The contribution of QT to the reaction rate grows at lower temperatures, and thus, the classical form of the TST rate constant expression is insufficient. A rigorous framework for incorporating the QT effect is provided by semiclassical instanton theory, which incorporates quantum tunneling effects naturally by identifying optimal tunneling paths in imaginary time, providing accurate rate constants across a wide range of temperatures.^56–61^ Another and simpler approach is to introduce QT correction with a correction coefficient. A quantum mechanical rate constant is then defined as *k*_TST_^QM^ = κ*k*_TST_, where κ is the QT correction coefficient and k_TST_ is the classical TST rate constant. The QT correction coefficients implemented in NAST were originally formulated by Wigner and Eckart.^62–65^ The Wigner correction reads as:

14 $$\kappa_{Wigner}=1+\frac{1}{24}{\left(\frac{h\left|\nu^{‡}\right|}{k_{B}T}\right)}^{2}$$

where ν^‡^ is the TS vibrational frequency. The Wigner correction is derived assuming a harmonic potential, where the transition state (TS) barrier is represented by an inverted parabola whose curvature, set by the force constant and expressed through the TS vibrational frequency, characterizes the barrier width. The Eckart correction, by contrast, is derived for a more realistic potential, which has the following form:

$$\begin{array}{l} V=-\frac{Ay}{1-y}-\frac{By}{{\left(1-y\right)}^{2}},\\ A=\Delta V_{1}-\Delta V_{2},\\ B={\left(\sqrt{\Delta V_{1}}+\sqrt{\Delta V_{2}}\right)}^{2},\\ y=-\exp(\frac{2\pi x}{L}),\\ L=2\pi \sqrt{-\frac{2}{k^{‡}}}\frac{1}{\left(\frac{1}{\Delta V_{1}}+\frac{1}{\Delta V_{2}}\right)} \end{array}$$ 15

where Δ*V*_1_ and Δ*V*_2_ are the potential energy barriers for the forward (reactant to product) and reverse (product to reactant) directions, respectively, *L* is the width of the potential, and *k* ^‡^ is the force constant associated with the TS vibrational mode. The Eckart correction is calculated as the convolution of the probability of QT through the TS potential barrier and the Boltzmann distribution of the system’s internal energy:

16 $$\begin{array}{l} \kappa_{Eckart}=\int_{\epsilon_{0}}^{\infty }P\left(V\right)e^{-\epsilon }d\epsilon ,\\ \epsilon =\{\begin{array}{ll} -\frac{\Delta V_{1}}{k_{B}T}, & \text{if}\;\Delta V_{1}\leq \Delta V_{2}\\ -\frac{\Delta V_{2}}{k_{B}T}, & \text{if}\;\Delta V_{1}>\Delta V_{2} \end{array} \end{array}$$

The Eckart probability of tunneling can be calculated analytically as:

17 $$P\left(V\right)=1-\frac{\cosh\left(2\pi \left(a-b\right)\right)+\cosh\left(2\pi d\right)}{\cosh\left(2\pi \left(a+b\right)\right)+\cosh\left(2\pi d\right)}$$

18 $$a=\frac{2\sqrt{V}}{h\left|\nu^{‡}\right|\left(\frac{1}{\Delta V_{1}}+\frac{1}{\Delta V_{2}}\right)}$$

19 $$b=\frac{2\sqrt{V-\Delta V_{1}+\Delta V_{2}}}{h\left|\nu^{‡}\right|\left(\frac{1}{\Delta V_{1}}+\frac{1}{\Delta V_{2}}\right)}$$

20 $$d=\frac{1}{2}\sqrt{\frac{16\Delta V_{1}\Delta V_{2}}{{\left(h\left|\nu^{‡}\right|\right)}^{2}}-1}$$

We have previously performed theoretical investigations of efficient C­(sp^2^)-H bond activation and functionalization catalyzed by the [Fe^*II*^(bqn)­(phen)­(Ph)­Cl]^0^ complex (bqn—a-benzoquinoline, phen—1,10- phenanthroline, Ph—phenyl) (Figure 5).^66^ It was proposed that the activation proceeds via a multi-state reaction sequence of quintet-triplet-singlet spin-forbidden transitions mediated by large spin–orbit couplings and results in the lowered effective reaction energy barrier. Here, we complement these studies by exploring the possibility of contribution from quantum tunneling through the TS energy barrier for the spin-allowed reaction step proceeding on the PES of the ground-state quintet, characterized by the (3d_*xy*_)^1^(3*d*_*xz*,*yz*_)^3^(3*d*_*z* ^2^_)^1^(3*d*_*x* ^2^‑*y* ^2^_)^1^ electronic configuration. Optimized geometries of the reactant, TS, and product were obtained at the B3LYP/def2-SVP level of theory, while rate constants were calculated using the energies refined at the ωB97X–V/def2-TZVP level of theory and simulating the solvent environment (THF) with the implicit CPCM solvation model. Electronic structure calculations were performed with the orca 6.0.1 program package.^43^ As shown in Figure 5, below room temperature, the quantum mechanical rate constant due to the Eckart QT correction is orders of magnitude larger than the semiclassical TST rate constant. This large QT correction originates from a large imaginary frequency ν^‡^ of 1477 cm^–1^ and, therefore, a narrow barrier at the TS geometry. This vibrational mode describes the transfer of the H atom from the benzoquinoline to phenyl, leading to the formation of the benzene molecule on the product’s side. Thus, QT could be of significant importance for the C­(sp^2^)-H bond activation by this Fe­(II) complex.

**Figure 5:**
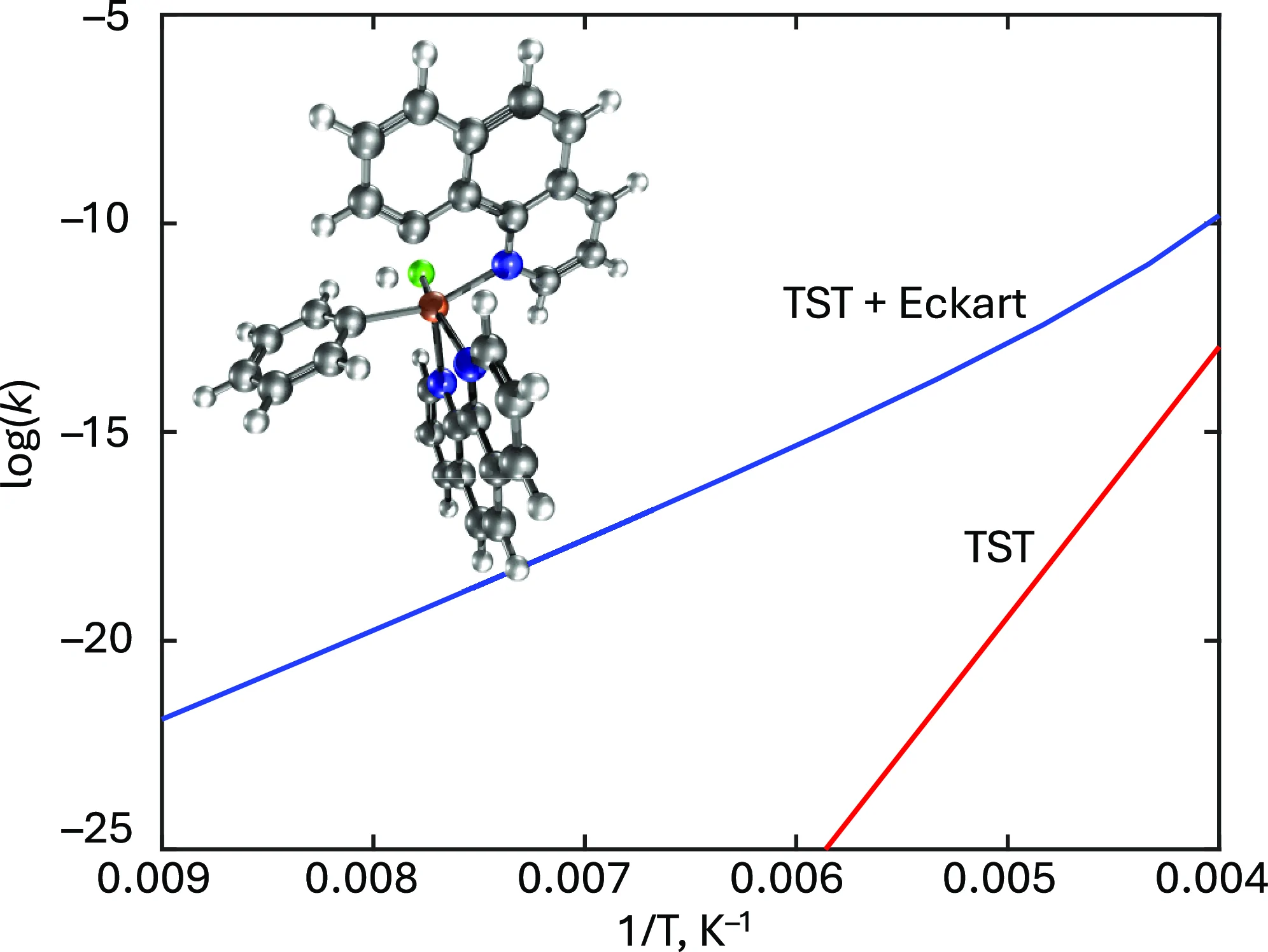
The log­(k) *vs*. 1/T plots for the semiclassical transition state theory rate constant (red) and quantum-mechanical rate constant obtained with the Eckart tunneling correction coefficient (blue). Inset: the optimized geometry of the quintet transition state. The Fe, Cl, N, C, and H atoms are shown in orange, green, blue, gray, and white, respectively. The shift of the H atom from the benzoquinoline to the phenyl moiety represents the normal mode of vibration at the transition state.

In principle, the tunneling correction can also be applied to the rate of the nonadiabatic transition. A lower-energy adiabatic potential can be constructed from adiabatization of the crossing potentials of two spin-diabatic states.^67^ Then, the tunneling coefficient can be calculated from the transition frequency at the saddle point on this adiabatic potential. This approach is useful for molecular systems with relatively strong SOC (typically a few hundred cm^–1^) such that the WC transition probability, which accounts for tunneling, becomes larger than one. In this scenario, the LZ rate constant with the Eckart correction, *k*_LZ_^QM^, can be used to account for quantum tunneling. Using a 1-D crossing model, we derived the expression for the force constant at the maximum of the lower adiabat and estimated the applicability limit of the adiabatization approach for the peaked-type intersection of parabolic potentials (see Supporting Information). We note that this approach for estimating nonadiabatic tunneling has limitations for more realistic crossings and should be used with care. In the present version of the nast package, the tunneling corrections are available only for the TST calculations. Calculation of the *k*_LZ_^QM^ rate constant would require two separate runs, one to obtain *k*_LZ_ with nast and the other to obtain κ_Wigner_ and κ_Eckart_ with TST.

### 2.5 MECP Search with pymecp

In this nast release, we included an MECP search program pymecp written in Python 3 and interfaced with the orca electronic structure package.^43^ This program is based on an earlier MECP search implementation by Harvey.^68^ It is capable of locating the MECP geometry between two spin states at any level of electronic structure theory, provided that energy gradients are available. Therefore, pymecp allows for the MECP search using the electronic structure methods for which the MECP optimization is not implemented internally in an electronic structure package. pymecp consists of two components: a Broyden–Fletcher–Goldfarb–Shanno (BFGS) optimization core and a wrapper script that manages the update and submission of electronic structure calculations. Details on how to use pymecp are provided in the nast manual.

### 2.6 Effective Hessian with Q-chem and Orca

In this version of nast, the *effhess* tool has been extended to work with the output files generated by the widely used orca 6 ^43^ and q-chem 5 ^69^ electronic structure packages, in addition to gamess^70^ and molpro^71^ as implemented earlier.^7^ The effective Hessian at MECP is constructed from the state-specific Hessians as:

21 $$H_{eff}=\frac{\left|g_{1}\right|H_{2}\pm \left|g_{2}\right|H_{1}}{\left|g_{1}\right|\pm \left|g_{2}\right|}$$

where **H**_1_ and **H**_2_ are state-specific Hessians calculated at the MECP geometry for states 1 and 2, respectively. The effective Hessian is then diagonalized to obtain the vibrational frequencies, moments of inertia, and the gradient norm |Δ**g**| and the mean gradient **g̅** at the MECP to calculate the probability of nonadiabatic transition as described in our previous work.^7,9^

### 2.7 IRC Fitting with Q-chem

An intrinsic reaction coordinate (IRC) calculation is used to locate a minimum energy path that connects the reactant’s and product’s minima to MECP. Molecular geometries and energies can then be expressed as a 1-D electronic potential along the reaction coordinate, which is useful for visualization and understanding of reaction dynamics. The explicit forms of the reaction potentials are also required for the calculations of transition probabilities using the ZN formulas. In contrast to the LZ and WC theories, which assume linear forms of the crossing potentials, the ZN formulation accounts for the nonlinear behavior of potential energy curves. The nast package is augmented with the *ircfit* tool to build the 1-D reaction potentials for spin-dependent reactions, provided with the IRC data from electronic structure calculations. Now, in addition to the gamess electronic structure package,^70^ nast also reads the IRC data from orca.

### 2.8 Adjustable Integration Step

We changed the previously fixed integration step of 1.0 cm^–1^ to be an adjustable parameter (eqs 1–5). An arbitrarily small integration step can now be specified, at the expense of increased computational cost. A smaller integration step may be required when performing rate calculations for small molecules. For molecules with only a few rovibrational degrees of freedom, the density of states is relatively sparse, and the convolution of the density of states with the transition probability can be more sensitive to the energy-grid discretization than for larger molecules. To estimate convergence of the rate calculations with respect to the integration step, we considered the examples of the isomerization of propylene oxide (ten atoms) and the triplet state relaxation in thiophosgene (four atoms).^7,8^ We found that for both processes, the rate constant readily converged within 1% at an integration step of 1.0 cm^–1^ (see Supporting Information). Thus, to ensure rate constant convergence, for small molecules we recommend performing several calculations with successively smaller integration steps, for example, 1.0, 0.5, and 0.25 cm^–1^. For larger molecules with many vibrational modes, the default integration step of 1.0 cm^–1^ is normally a sufficient compromise due to the high density of rovibrational states involved in integration. While the main error in the rate constant often arises from the limited accuracy of electronic-structure calculations, the new integration step-size control can help eliminate numerical integration error in the rate equation. This is especially relevant for reactions involving small molecules containing only a few atoms, where highly accurate electronic structure calculations are feasible, and rate constants can be computed with correspondingly high accuracy.

## 3 Conclusions

The nast package is a powerful tool for predicting the kinetics of spin-dependent processes, such as intersystem crossings, spin-forbidden reactions, and spin crossovers. In this work, we presented a new release of nast, which can now be applied to predict spin-dependent kinetics in external magnetic fields, perform Marcus–Levich–Jortner (MLJ) theory calculations to study the kinetics in complex open-shell systems, and calculate quantum tunneling corrections to the semiclassical transition state rate constants. This release also significantly expands the technical capabilities of nast. The newly added minimum-energy crossing point (MECP) search program pymecp interfaced with  orca allows for locating MECP geometries with various electronic structure methods for which MECP optimization is not yet supported. A new *vib* tool allows for calculation of Huang–Rhys factors and reorganization energies used in MLJ calculations. nast was also extended to support the effective Hessian calculations at the MECP with orca and q-chem packages and the intrinsic reaction coordinate fit with orca. Furthermore, we implemented integration step-size control. This allows for higher integration accuracy, thereby facilitating more accurate computations of the rate constants. Collectively, these enhancements significantly broaden the application scope of nast, enabling it to predict the kinetics of a diverse range of nonadiabatic and adiabatic processes (Figure 6).

**Figure 6:**
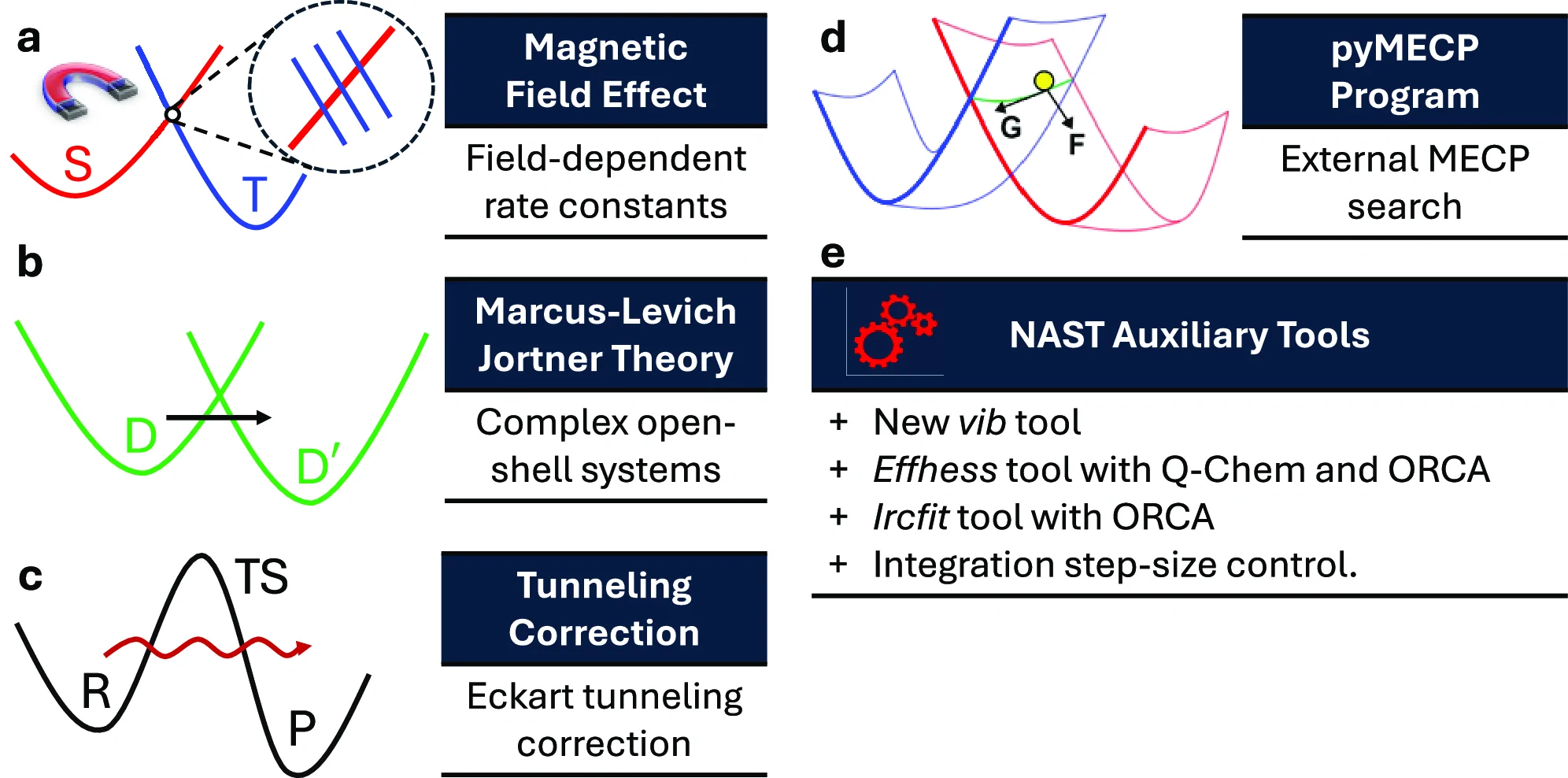
Summary of the new capabilities of the nast package. (a) Magnetic field effect calculations with NAST. (b) New Marcus–Levich–Jortner theory program mlj for modeling kinetics in complex open-shell systems. (c) Eckart tunneling coefficient for correcting the TST rate constants. (d) New pymecp program for locating the MECP geometries. (e) A set of new and extended auxiliary tools to facilitate the mlj and nast calculations. In panels *a*, *b*, and *c*, *S*, *T*, and *D* denote singlet, triplet, and doublet spin states, respectively. *R*, *P*, and *TS* stand for reactant, product, and transition state, respectively. In panel *d*, *G* and *F* are the two gradient vectors to be minimized.

As a next step, both the MLJ theory and the new capability of nast to calculate transition probabilities and rates in external magnetic fields naturally motivate the prediction of field-dependent spin sublevel population dynamics. Each approach provides a pathway for modeling spin polarization, which is of particular importance in dynamic nuclear polarization for magnetic resonance signal enhancement and quantum sensing.

## Supplementary Material



## Data Availability

The nast package is available free of charge at https://github.com/svarganov/NAST/ together with a detailed user manual.

## Notes

Structure of the NAST package, MLJ calculations for TTM-1Cz-An, TST calculations, integration step size information, and XYZ coordinates used in calculations (PDF)
