# Supplementary material for: NAST 2.0: Magnetic Field Effects and Marcus–Levich–Jortner Theory for Complex Open-Shell Systems
Source: ACS Omega. 2026 Sep 20;11(39):59936–45. doi: 10.1021/acsomega.6c07270 (PMC13647926; doi:10.1021/acsomega.6c07270)
Supplement: Supplementary file 1 [file ao6c07270_si_001.pdf]

NAST 2.0: Magnetic Field Effects and Marcus-Levich-Jortner Theory for Complex Open-Shell Systems
Supplementary Information

Ilya D. Dergachev,[†] Vsevolod D. Dergachev,[‡] Mitra Rooein,[¶] Yash H. Patel,[†] Philip S. Weiss,[†] Claudia E. Avalos,[*,†] and Sergey A. Varganov[*,‡]

†*Molecular Design Institute, Department of Chemistry, New York University, New York City, New York 10003, United States*

‡*Department of Chemistry, University of Nevada, Reno, Reno 89557-0216, United States*

¶*Department of Chemistry for Life Sciences, Uppsala University, Uppsala, Sweden*

E-mail: claudia.avalos@nyu.edu; svarganov@unr.edu

Contents

1 Structure of the NAST package **3**

2 MLJ calculations for TTM-1Cz-An **4**

3 TST calculations for $[Fe^{II}(bqn)(phen)(Ph)Cl]^0$ **18**

4 Estimating the curvature of the MECP barrier **25**

5 Integration step size **29**

6 XYZ coordinates **30**

1 Structure of the NAST package

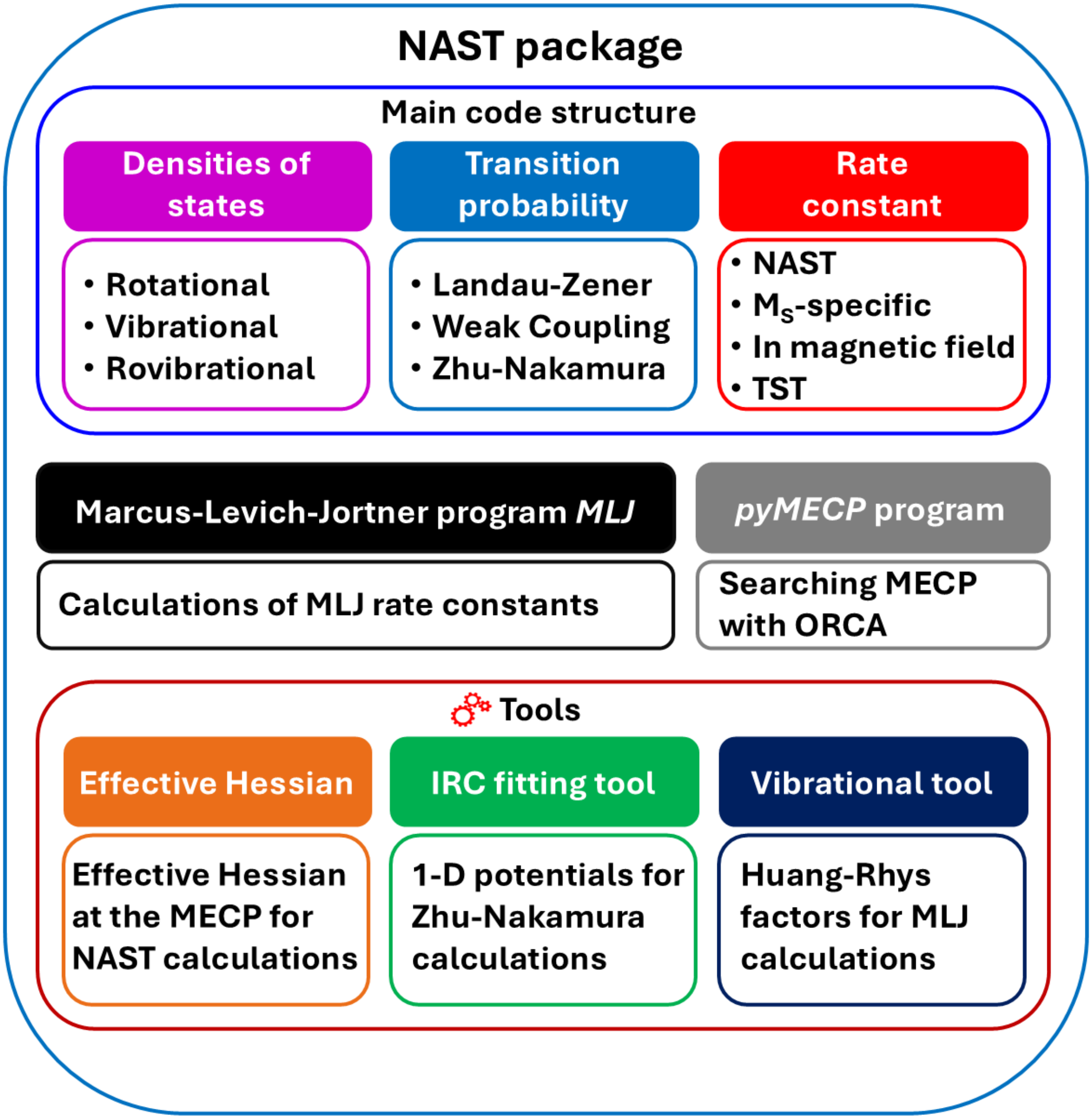

Figure S1: The structure of the NAST package. The top panel shows the workflow of the main code. The central panel shows the PYMECP program for the minimum-energy crossing point search with ORCA and the MLJ program for calculating the Marcus-Levich-Jortner rate constants. The bottom panel shows the additional programs called tools used to simplify the use of the NAST package.

The NAST package is written in the modern Fortran program language. It is designed to run on Linux and has a modular structure, as shown in Figure S1. In addition to the main code, the package is supplemented with the auxiliary programs called tools, which allow for collecting a part of the data required for *NAST* calculations. The effective Hessian tool *effhess* computes the effective Hessian at the MECP, extracts the vibrational data, and creates the NAST template. The intrinsic reaction coordinate fitting tool *ircfit* builds 1-D crossing potentials for advanced Zhu-Nakamura calculations. The vibrational tool *vib* computes the Huang-Rhys factors and reorganization energies for calculating the Marcus-Levich-Jortner theory rate constants. The NAST package is distributed with a detailed user manual that covers installation, execution, and analysis of the *NAST* results. The manual also provides a full list of keywords and their default values. The pyMECP program is distributed together with the *NAST* package and is written in Python 3 program language. The pyMECP program is interfaced with the ORCA[1] electronic structure program and is designed to search for the MECP geometries at levels of theory that currently lack the MECP optimization procedures. More details on the NAST structure and workflow are given in our first release paper.[2] The new capabilities introduced in this work are discussed in the main text.

2 MLJ calculations for TTM-1Cz-An

The active space made of nine electrons in seven orbitals was chosen from the previous multireference calculations on the TTM-1Cz-An radical,[3] and the active space orbitals are shown in Figure S2. The state-average CASSCF calculations included one quartet state and seven doublet states.

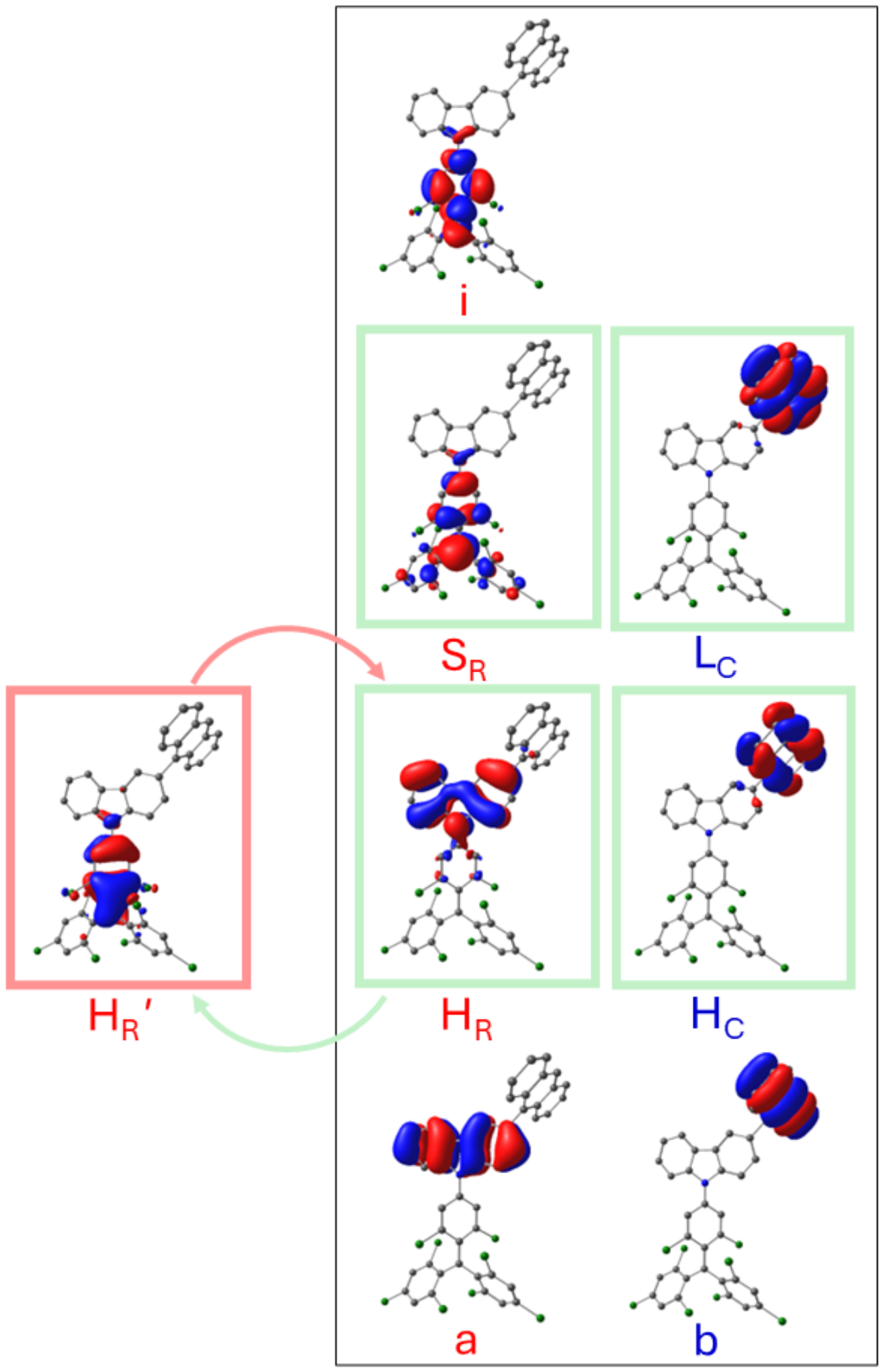

Figure S2: The CASSCF molecular orbitals of the TTM-1Cz-An radical. The active space orbitals are shown in the black box with the four main orbitals shown in green boxes. These four orbitals are the TTM-1Cz radical's HOMO (H_R) and SOMO (S_R) and anthracene chromophore's orbitals HOMO (H_C) and LUMO (L_C). The role of another occupied radical's orbital ($H^{'}_R$) is explained in the text.

At the geometry of the $^2[D_1S_0]$ state, we were not able to keep the H_R orbital inside the active space, and it was replaced with the $H^{'}_R$ orbital due to a lower total CASSCF energy because of the variational nature of the CASSCF method. Thus, due to the missing H_R orbital, the $^2[D_1S_0]$ state could not be predicted. The energy of the $^2[D_1S_0]$ state at its equilibrium geometry was obtained by rotating H_R and $H^{'}_R$ orbitals and performing CASSCF/NEVPT2 calculation without a new CASSCF optimization procedure. As for the

CASSCF calculations at the $^2[D_0T_1]$ geometry, the orbital b was replaced with the H'_R orbital during the CASSCF optimization, and this one orbital-different active space gave the energy of the $^2[D_0T_1]$ state 28 meV lower than the energy of the $^2[D_1S_0]$ as reported in the main text.

Table S1: Energy gap (ΔE), electronic coupling between initial and final states (V_{if}), inner (λ_{vib}) and outer (λ_{ext}) reorganization energies, and effective frequency ($\hbar\omega$) used to calculate the Marcus–Levich–Jortner rate constant (k) in this and a previous work.[3]

	ΔE, meV	V_{if}, meV	λ_{vib}, eV	λ_{ext}, eV	$\hbar\omega$, eV	k, s^{-1}
This work	28	5	0.80		0.18	$2.7 \cdot 10^9$
Previous work	20	5	0.60	0.10	0.15	$6.6 \cdot 10^9$

Input file for calculating the Marcus-Levich-Jortner rate constant with the MLJ program:

```
&inputdata
enR = 0.0d0      ! Reactant energy.
enP = 0.00104048 ! Product energy in hartree.
V = 40.3 ! Coupling constant in cm-1.
freR = <array of vibrational frequencies from Table S2 below> ! In cm-1.
S = <array of Huang-Rhys factors from Table S2 below>
lambda_vib = <array of vibrational reorganization energies from Table S2 below>
                 ! In hartree.
lambda_ext = 0.00d0
n_vib = 10       ! n in Eq. 10 from the main text.
T1 = 250         ! Temperature in K.
T2 = 300
Tstep = 10
trsh = 4.796     ! Threshold between classical and quantum modes.
&end
```

Table S2: Vibrational modes, Huang–Rhys factors and reorganization energies calculated from the minima of $^2[D_1S_0]$ and $^2[D_0T_1]$ states and the $^2[D_1S_0]$ Hessian.

ν (cm^{-1})	S	λ_{vib}
6.4	0.5325	1.546×10^{-5}
7.0	1.288×10^{-4}	4.109×10^{-9}
12.3	0.6399	3.588×10^{-5}
21.8	0.1446	1.433×10^{-5}
26.3	0.1858	2.225×10^{-5}
28.2	0.2573	3.307×10^{-5}
31.8	1.315×10^{-2}	1.905×10^{-6}
35.3	2.491×10^{-2}	4.008×10^{-6}
39.9	2.089	3.799×10^{-4}
52.7	2.035	4.885×10^{-4}
54.1	10.67	2.629×10^{-3}
61.2	0.1993	5.560×10^{-5}
68.8	4.212×10^{-3}	1.320×10^{-6}
82.7	0.4250	1.601×10^{-4}
86.2	0.3771	1.481×10^{-4}
88.8	8.886×10^{-2}	3.595×10^{-5}
105.6	9.210×10^{-3}	4.432×10^{-6}
116.4	2.901×10^{-2}	1.538×10^{-5}
125.4	4.001×10^{-3}	2.285×10^{-6}
138.3	1.378×10^{-2}	8.683×10^{-6}
146.7	0.3514	2.349×10^{-4}
148.8	1.048×10^{-4}	7.106×10^{-8}
150.2	5.135×10^{-3}	3.513×10^{-6}
155.5	0.1386	9.817×10^{-5}

ν (cm^{-1})	S	λ_{vib}
165.0	5.451×10^{-3}	4.097×10^{-6}
166.9	6.497×10^{-3}	4.939×10^{-6}
181.6	3.520×10^{-3}	2.912×10^{-6}
193.1	3.040×10^{-4}	2.674×10^{-7}
197.4	3.725×10^{-3}	3.351×10^{-6}
201.9	9.643×10^{-4}	8.870×10^{-7}
212.4	3.113×10^{-4}	3.012×10^{-7}
214.3	5.809×10^{-2}	5.673×10^{-5}
228.8	1.465×10^{-2}	1.528×10^{-5}
233.9	9.493×10^{-2}	1.012×10^{-4}
239.1	0.1979	2.156×10^{-4}
241.5	0.2927	3.220×10^{-4}
242.6	0.2804	3.098×10^{-4}
263.2	3.751×10^{-2}	4.499×10^{-5}
272.1	6.452×10^{-2}	7.998×10^{-5}
295.9	4.372×10^{-3}	5.894×10^{-6}
316.3	8.215×10^{-3}	1.184×10^{-5}
331.3	9.439×10^{-3}	1.425×10^{-5}
337.5	3.271×10^{-2}	5.029×10^{-5}
355.4	1.705×10^{-2}	2.760×10^{-5}
362.2	3.613×10^{-3}	5.962×10^{-6}
369.0	7.208×10^{-2}	1.212×10^{-4}
380.4	3.751×10^{-2}	6.500×10^{-5}
380.8	0.1866	3.238×10^{-4}
403.4	0.2731	5.019×10^{-4}
406.4	2.521×10^{-3}	4.669×10^{-6}

ν (cm^{-1})	S	λ_{vib}
428.6	9.407×10^{-4}	1.837×10^{-6}
432.9	9.656×10^{-4}	1.905×10^{-6}
435.2	6.210×10^{-2}	1.231×10^{-4}
438.5	1.683×10^{-3}	3.363×10^{-6}
441.7	2.074×10^{-4}	4.174×10^{-7}
444.5	3.045×10^{-5}	6.168×10^{-8}
445.2	5.981×10^{-3}	1.213×10^{-5}
448.1	4.804×10^{-4}	9.808×10^{-7}
462.7	1.765×10^{-4}	3.720×10^{-7}
467.8	8.703×10^{-3}	1.855×10^{-5}
483.9	1.520×10^{-2}	3.350×10^{-5}
503.7	1.040×10^{-3}	2.387×10^{-6}
520.2	1.368×10^{-4}	3.242×10^{-7}
524.7	1.759×10^{-5}	4.205×10^{-8}
545.6	2.392×10^{-3}	5.947×10^{-6}
547.1	5.677×10^{-3}	1.415×10^{-5}
549.5	2.234×10^{-3}	5.593×10^{-6}
557.0	2.723×10^{-2}	6.910×10^{-5}
559.9	1.664×10^{-3}	4.245×10^{-6}
564.7	2.092×10^{-5}	5.383×10^{-8}
570.3	1.298×10^{-2}	3.371×10^{-5}
574.9	5.296×10^{-6}	1.387×10^{-8}
586.1	1.103×10^{-4}	2.944×10^{-7}
589.7	7.183×10^{-5}	1.930×10^{-7}
593.7	2.349×10^{-4}	6.355×10^{-7}
616.9	2.950×10^{-4}	8.292×10^{-7}

ν (cm^{-1})	S	λ_{vib}
620.9	1.112×10^{-4}	3.145×10^{-7}
628.5	3.804×10^{-4}	1.089×10^{-6}
636.3	2.180×10^{-4}	6.321×10^{-7}
655.6	2.659×10^{-2}	7.942×10^{-5}
666.1	1.730×10^{-4}	5.251×10^{-7}
671.6	3.148×10^{-3}	9.631×10^{-6}
678.3	8.829×10^{-5}	2.729×10^{-7}
688.0	1.741×10^{-6}	5.456×10^{-9}
712.2	4.505×10^{-3}	1.462×10^{-5}
725.5	8.015×10^{-4}	2.649×10^{-6}
754.3	4.946×10^{-3}	1.700×10^{-5}
772.8	5.685×10^{-7}	2.002×10^{-9}
774.6	9.809×10^{-5}	3.462×10^{-7}
778.6	1.372×10^{-4}	4.869×10^{-7}
780.2	9.194×10^{-6}	3.268×10^{-8}
794.2	9.183×10^{-4}	3.323×10^{-6}
797.1	1.057×10^{-5}	3.838×10^{-8}
800.0	1.459×10^{-6}	5.320×10^{-9}
804.3	9.347×10^{-3}	3.425×10^{-5}
808.3	3.087×10^{-5}	1.137×10^{-7}
821.1	3.109×10^{-4}	1.163×10^{-6}
826.7	2.634×10^{-3}	9.922×10^{-6}
828.6	3.296×10^{-2}	1.244×10^{-4}
842.1	1.403×10^{-5}	5.384×10^{-8}
847.5	1.228×10^{-3}	4.740×10^{-6}
850.0	8.337×10^{-3}	3.229×10^{-5}

ν (cm^{-1})	S	λ_{vib}
856.1	5.179×10^{-4}	2.020×10^{-6}
878.1	7.392×10^{-4}	2.957×10^{-6}
886.3	9.811×10^{-6}	3.962×10^{-8}
903.9	4.230×10^{-6}	1.742×10^{-8}
905.2	5.719×10^{-5}	2.359×10^{-7}
911.4	2.217×10^{-4}	9.209×10^{-7}
912.6	3.030×10^{-5}	1.260×10^{-7}
913.3	1.142×10^{-3}	4.754×10^{-6}
919.9	6.623×10^{-5}	2.776×10^{-7}
921.0	1.366×10^{-4}	5.733×10^{-7}
924.6	2.623×10^{-3}	1.105×10^{-5}
928.1	1.024×10^{-2}	4.332×10^{-5}
942.5	6.486×10^{-4}	2.785×10^{-6}
945.1	0.1534	6.606×10^{-5}
948.9	4.442×10^{-5}	1.920×10^{-7}
955.9	6.945×10^{-4}	3.025×10^{-6}
965.6	6.762×10^{-5}	2.975×10^{-7}
968.4	8.158×10^{-5}	3.599×10^{-7}
974.7	1.417×10^{-4}	6.295×10^{-7}
1002.0	2.386×10^{-4}	1.089×10^{-6}
1015.5	1.245×10^{-3}	5.762×10^{-6}
1016.6	0.08439	3.909×10^{-4}
1022.5	1.008×10^{-3}	4.697×10^{-6}
1025.4	1.605×10^{-4}	7.498×10^{-7}
1043.7	9.873×10^{-7}	4.695×10^{-9}
1048.4	3.404×10^{-2}	1.626×10^{-4}

ν (cm^{-1})	S	λ_{vib}
1048.9	9.938×10^{-4}	4.749×10^{-6}
1049.2	1.122×10^{-2}	5.365×10^{-5}
1050.6	4.347×10^{-4}	2.081×10^{-6}
1051.5	1.124×10^{-3}	5.387×10^{-6}
1061.4	1.233×10^{-3}	5.965×10^{-6}
1080.1	2.368×10^{-2}	1.166×10^{-4}
1100.1	5.639×10^{-2}	2.826×10^{-4}
1106.6	3.038×10^{-4}	1.532×10^{-6}
1136.5	1.144×10^{-2}	5.926×10^{-5}
1138.7	1.254×10^{-4}	6.508×10^{-7}
1153.3	3.091×10^{-4}	1.624×10^{-6}
1154.0	7.741×10^{-4}	4.070×10^{-6}
1157.6	1.621×10^{-5}	8.548×10^{-8}
1164.2	4.939×10^{-3}	2.620×10^{-5}
1167.2	3.272×10^{-5}	1.740×10^{-7}
1169.4	1.076×10^{-5}	5.734×10^{-8}
1178.3	0.1236	6.636×10^{-4}
1193.6	2.038×10^{-5}	1.108×10^{-7}
1198.3	7.800×10^{-4}	4.259×10^{-6}
1201.5	2.203×10^{-4}	1.206×10^{-6}
1203.6	1.201×10^{-2}	6.587×10^{-5}
1205.4	4.490×10^{-4}	2.466×10^{-6}
1214.4	6.779×10^{-2}	3.751×10^{-4}
1229.7	6.783×10^{-2}	3.801×10^{-4}
1247.7	1.756×10^{-5}	9.984×10^{-8}
1249.7	3.441×10^{-3}	1.959×10^{-5}

ν (cm^{-1})	S	λ_{vib}
1273.1	3.997×10^{-2}	2.319×10^{-4}
1287.1	4.758×10^{-5}	2.790×10^{-7}
1295.8	1.870×10^{-2}	1.104×10^{-4}
1296.6	2.355×10^{-4}	1.391×10^{-6}
1297.4	0.2071	1.224×10^{-3}
1304.8	0.0995	5.915×10^{-4}
1317.9	0.1193	7.165×10^{-4}
1325.5	0.1745	1.054×10^{-3}
1340.5	3.933×10^{-3}	2.402×10^{-5}
1344.1	1.465×10^{-4}	8.970×10^{-7}
1345.9	1.977×10^{-2}	1.212×10^{-4}
1357.6	5.997×10^{-4}	3.709×10^{-6}
1383.3	1.426×10^{-3}	8.988×10^{-6}
1405.6	3.167×10^{-6}	2.028×10^{-8}
1411.5	5.678×10^{-2}	3.652×10^{-4}
1415.3	2.149×10^{-4}	1.386×10^{-6}
1429.4	1.821×10^{-2}	1.186×10^{-4}
1432.6	1.175×10^{-4}	7.673×10^{-7}
1439.2	2.008×10^{-4}	1.316×10^{-6}
1446.6	1.445×10^{-6}	9.523×10^{-9}
1449.0	0.1293	8.534×10^{-4}
1478.0	5.587×10^{-2}	3.762×10^{-4}
1485.1	1.394×10^{-2}	9.430×10^{-5}
1489.5	0.4112	2.790×10^{-3}
1493.5	1.116×10^{-4}	7.592×10^{-7}
1506.7	1.746×10^{-3}	1.199×10^{-5}

ν (cm^{-1})	S	λ_{vib}
1510.7	1.746×10^{-3}	1.202×10^{-5}
1516.5	1.534×10^{-2}	1.060×10^{-4}
1522.4	7.149×10^{-2}	4.959×10^{-4}
1531.4	4.574×10^{-2}	3.192×10^{-4}
1549.1	1.826×10^{-2}	1.289×10^{-4}
1555.1	3.539×10^{-4}	2.508×10^{-6}
1586.8	1.129×10^{-6}	8.160×10^{-9}
1591.6	1.052×10^{-6}	7.632×10^{-9}
1608.9	6.222×10^{-6}	4.561×10^{-8}
1627.6	9.184×10^{-5}	6.811×10^{-7}
1646.0	2.164×10^{-4}	1.623×10^{-6}
1654.4	0.5347	4.031×10^{-3}
1657.2	0.1527	1.153×10^{-3}
1663.2	7.386×10^{-5}	5.598×10^{-7}
1670.5	2.383×10^{-4}	1.814×10^{-6}
1682.6	2.351×10^{-3}	1.802×10^{-5}
1698.6	0.1386	1.073×10^{-3}
1716.6	0.3895	3.046×10^{-3}
1722.1	2.182×10^{-4}	1.712×10^{-6}
1723.8	1.138×10^{-3}	8.935×10^{-6}
3196.9	3.517×10^{-4}	5.122×10^{-6}
3198.4	4.254×10^{-6}	6.200×10^{-8}
3199.7	1.928×10^{-4}	2.811×10^{-6}
3206.8	6.458×10^{-5}	9.437×10^{-7}
3207.8	1.459×10^{-4}	2.132×10^{-6}
3208.5	1.218×10^{-5}	1.781×10^{-7}

ν (cm^{-1})	S	λ_{vib}
3213.3	1.171×10^{-5}	1.715×10^{-7}
3215.2	5.076×10^{-6}	7.435×10^{-8}
3217.1	3.462×10^{-7}	5.074×10^{-9}
3219.6	2.023×10^{-7}	2.968×10^{-9}
3219.8	1.511×10^{-3}	2.217×10^{-5}
3227.5	6.053×10^{-6}	8.901×10^{-8}
3231.4	8.241×10^{-5}	1.213×10^{-6}
3232.2	5.120×10^{-4}	7.540×10^{-6}
3240.8	7.250×10^{-6}	1.071×10^{-7}
3241.0	1.275×10^{-4}	1.883×10^{-6}
3245.2	1.324×10^{-3}	1.957×10^{-5}
3246.4	5.329×10^{-4}	7.883×10^{-6}
3247.1	2.026×10^{-3}	2.998×10^{-5}
3247.3	2.039×10^{-3}	3.017×10^{-5}
3247.7	1.013×10^{-4}	1.499×10^{-6}
3247.9	5.033×10^{-4}	7.448×10^{-6}

All electronic structure calculations on the TTM-1Cz-An radical have been performed with the ORCA 6.1 electronic structure package.[1] Input file for the $^2[D_1S_0]$ state geometry optimization:

```
! UKS CAM-B3LYP def2-SVP verytightscf Opt Freq CPCM(Toluene) moread
%maxcore 8000
%pal nprocs 40 end
%moinp "start_TDA.gbw"
%tddft
  nroots 2
```

```
  iroot 2 end
%output
  Print[P_basis] 2
  Print[P_MOs] 1
end
* xyz 0 2
Starting XYZ coordinates
*
```

Input file for the $^4[D_0T_1]$ state geometry optimization:

```
! UKS CAM-B3LYP def2-SVP verytightscf Opt Freq CPCM(Toluene) moread
%maxcore 8000
%pal nprocs 40 end
%moinp "start_DFT.gbw"
%output
  Print[P_basis]2
  Print[P_MOs]1
end
* xyz 0 4
Starting XYZ coordinates
*
```

Example of an input file for the CASSCF/NEVPT2 calculations:

```
!RI-JK def2-TZVP verytightscf moread noiter
 %maxcore 8000
 %pal nprocs 40 end
 %moinp "2D1S0_cas9e7o_tzvp_7D1Q.gbw"
```

```
 %scf rotate {212, 213, 90, 0, 0} end
  end
 %basis
  auxC "def2-TZVP/C"
  auxJK "def2/JK"
 end
 %casscf
  trafostep ri
  nel 9
  norb 7
  mult 2,4
  nroots 7,1
   weights[0]=1,1,1,1,1,1,1
   weights[1]=1
  PrintWF det
  ActOrbs CanonOrbs
  PTMethod SC_NEVPT2
 end
 %output
   Print[P_basis]2
   Print[P_MOs]1
 end
* xyz 0 2
Optimzed XYZ coordinates
*
```

3 TST calculations for $[Fe^{II}(bqn)(phen)(Ph)Cl]^0$

The NAST input file for calculating Eckart quantum tunneling correction:

```
&keys zpe = 1 tst = .true. TST_tunn=.t. rev=.t. printmore = .t. &end

&inputdata
TST_freq = 1476  ! Real part of the TS frequqncy in cm-1.
freX = <>        ! Array of TS frequencies (Table S3).
freR = <>        ! Array of reactant frequencies (Table S3).
inertX = 12330.99827 19763.60261 22107.94475
inertR = 13365.88223 19763.60261 22699.82411
enX = -3082.7367216d0
enR = -3082.7877653d0
maxn = 20000
T1 = 100
Tstep = 10
T2 = 300
&end

&reverse
freP =   <>  ! Not required for Eckart QT correction, can be copied from freR.
inertP = <>  ! Not required for Eckart QT correction, can be copied from inertR.
enP = -3082.7898235d0
&end
```

Table S3: Vibrational frequencies (cm^{-1}) for reactant (R), transition state (TS), and product (P) states of the $[Fe^{II}$(bqn)(phen)(Ph)Cl$]^0$ complex. Benzene (Ph-H) is another product of the reaction.

R	TS	P	Ph-H
10.6	18.9	16.6	418.5
13.2	19.6	23.6	418.8
19.6	26.7	29.1	620.2
25.2	30.9	36.4	620.3
30.0	44.1	79.9	694.4
31.5	50.1	81.7	733.9
48.0	72.0	91.1	870.7
59.5	77.9	111.4	871.2
73.0	86.3	128.5	1004.4
80.2	96.3	133.5	1004.7
96.5	109.4	145.8	1014.0
108.0	129.3	156.6	1018.3
115.0	135.3	177.5	1040.2
125.9	143.1	200.3	1057.5
133.5	154.5	233.6	1057.6
142.6	160.0	241.8	1161.5
149.5	161.9	244.2	1183.6
157.4	171.4	259.8	1183.8
170.0	204.1	266.7	1362.8
187.5	208.7	286.6	1365.7
233.3	231.8	290.4	1508.6
240.8	242.1	332.6	1508.8
241.8	242.8	422.0	1650.0

R	TS	P	Ph-H
251.9	263.3	426.7	1650.4
260.1	270.4	427.4	3162.6
266.9	284.5	429.1	3172.2
277.1	290.2	458.9	3172.4
288.9	310.3	474.7	3186.6
402.9	407.3	481.3	3186.9
412.6	421.1	487.1	3196.0
421.9	422.9	514.1	
422.8	427.0	518.5	
427.6	432.4	520.3	
448.2	461.0	523.6	
454.6	468.8	562.5	
458.5	471.1	564.8	
472.2	485.4	570.9	
483.0	491.0	578.4	
512.1	516.3	625.4	
518.0	517.1	627.9	
519.1	519.3	638.0	
521.8	525.6	652.2	
558.1	559.9	730.7	
565.2	564.9	732.1	
571.5	572.3	735.8	
572.6	579.7	745.8	
618.4	622.3	756.6	
629.1	628.7	756.9	
631.6	630.2	776.9	

R	TS	P	Ph-H
636.9	642.0	789.2	
644.5	643.0	807.2	
655.3	660.3	823.1	
724.5	725.2	863.1	
725.5	733.0	870.3	
730.5	733.9	874.3	
731.2	736.6	874.7	
736.8	742.1	875.6	
747.3	754.7	878.3	
753.9	756.9	905.6	
759.3	760.8	916.2	
770.6	779.5	935.1	
788.5	788.7	963.3	
808.2	808.9	984.6	
824.3	822.3	988.5	
844.9	864.8	1002.3	
855.0	866.2	1010.3	
873.1	874.1	1013.6	
873.6	876.0	1017.6	
875.3	877.3	1025.1	
876.9	880.7	1028.3	
891.0	882.8	1030.1	
897.5	908.7	1051.5	
907.9	915.4	1071.8	
918.9	941.0	1073.1	
934.2	944.0	1094.9	

R	TS	P	Ph-H
974.0	960.4	1103.6	
975.6	974.9	1114.2	
995.8	992.5	1117.1	
996.4	1004.2	1149.0	
1004.1	1004.7	1150.3	
1006.1	1008.7	1154.8	
1008.8	1012.5	1157.9	
1013.4	1013.2	1208.9	
1017.8	1021.2	1222.7	
1022.0	1021.6	1233.5	
1026.1	1026.4	1238.1	
1032.3	1028.5	1241.3	
1033.0	1032.0	1242.4	
1035.3	1037.3	1263.3	
1041.7	1051.4	1281.6	
1051.1	1067.5	1335.7	
1064.6	1071.7	1343.3	
1072.5	1073.8	1346.4	
1076.0	1079.3	1372.6	
1080.4	1102.9	1374.0	
1086.0	1105.6	1376.9	
1104.7	1115.8	1423.6	
1113.9	1119.3	1430.8	
1115.5	1149.6	1436.2	
1149.2	1151.1	1436.9	
1149.9	1155.4	1444.8	

R	TS	P	Ph-H
1155.1	1160.6	1455.6	
1161.7	1163.1	1465.7	
1161.8	1169.5	1480.5	
1173.2	1193.3	1530.2	
1197.6	1208.4	1533.3	
1222.3	1224.4	1535.9	
1229.3	1233.0	1550.6	
1236.3	1242.1	1603.0	
1240.8	1242.2	1615.2	
1242.4	1243.0	1623.4	
1245.2	1267.4	1632.3	
1285.7	1285.1	1646.3	
1293.4	1334.4	1654.2	
1298.1	1335.6	1662.1	
1338.1	1345.0	1669.5	
1344.8	1345.8	3127.1	
1346.4	1349.9	3154.7	
1366.9	1370.4	3159.9	
1375.4	1374.1	3169.9	
1376.0	1377.0	3173.2	
1394.2	1417.1	3173.8	
1430.4	1436.7	3174.1	
1436.7	1436.9	3174.7	
1438.8	1439.4	3179.4	
1442.5	1444.7	3179.9	
1443.3	1457.3	3180.5	

R	TS	P	Ph-H
1457.9	1458.9	3184.9	
1475.0	1470.2	3190.7	
1479.1	1480.6	3202.2	
1480.3	1498.5	3205.4	
1494.3	1532.2	3206.5	
1534.8	1533.9		
1537.3	1548.2		
1549.6	1552.6		
1562.4	1607.3		
1608.8	1617.2		
1618.4	1617.5		
1618.9	1625.4		
1620.9	1630.0		
1635.9	1632.9		
1643.4	1651.9		
1656.1	1654.0		
1661.2	1669.1		
1669.8	1670.8		
1673.5	1751.4		
3119.6	3133.1		
3139.1	3142.3		
3147.3	3150.2		
3155.9	3157.8		
3161.4	3162.0		
3167.4	3164.8		
3169.9	3167.8		

R	TS	P	Ph-H
3172.2	3170.9		
3172.9	3173.8		
3174.8	3174.7		
3177.2	3178.1		
3179.9	3178.2		
3180.5	3179.3		
3185.1	3180.2		
3186.6	3180.8		
3188.0	3183.7		
3191.3	3187.2		
3198.7	3191.2		
3204.5	3206.1		
3206.8	3206.8		
3208.0	3207.2		
3208.7			

The ORCA input files for the DFT geometry optimization for the $[Fe^{II}(bqn)(phen)(Ph)Cl]^0$ complex are very similar to the input file for the DFT geometry optimization for TTM-1Cz-An.

4 Estimating the curvature of the MECP barrier

Here we derive an expression for the force constant associated with a TS-like barrier on the lower of two potentials obtained from the adiabatization of two spin diabats at the minimum energy crossing point (MECP). For illustration, we use the 1-D model of two

crossing parabolic potentials.

The force constant describing the curvature of the MECP barrier formed by two intersecting spin diabats can be obtained by diagonalizing the 2×2 matrix:

$$\begin{pmatrix} V_1 & H_{\mathrm{SO}} \\ H_{\mathrm{SO}} & V_2 \end{pmatrix}. \tag{S1}$$

where V_1 and V_2 are the energies of spin-diabatic states at the MECP, and H_{SO} is the spin-orbit coupling constant calculated as:

$$H_{\mathrm{SO}} = \left(\sum_{M_S} \sum_{M'_S} \left| \left\langle S, M_S \middle| \widehat{H}_{\mathrm{SO}} \middle| S', M'_S \right\rangle \right|^2 \right)^{1/2}. \tag{S2}$$

where M_S and M'_S are the components of the S and S' spin states, respectively.

Solving the eigenvalue problem for equation S3, we obtain the energy of the lower spin-adiabatic state V_{ad}:

$$V_{\mathrm{ad}} = \frac{1}{2}(V_1 + V_2) - \frac{1}{2}\sqrt{(V_1 - V_2)^2 + 4H_{\mathrm{SO}}^2}. \tag{S3}$$

The force constant is the second derivative of energy:

$$\frac{dV_{\mathrm{ad}}}{dx} = \frac{1}{2}\left(\frac{dV_1}{dx} + \frac{dV_2}{dx}\right) - \frac{1}{4}C, \tag{S4}$$

where

$$\begin{aligned} C = \; & \left[(V_1 - V_2)^2 + 4H_{\rm SO}^2\right]^{-1/2} \\ & \times \left[2(V_1 - V_2)\left(\frac{dV_1}{dx} - \frac{dV_2}{dx}\right) + 8H_{\rm SO}\frac{dH_{\rm SO}}{dx}\right]. \end{aligned} \tag{S5}$$

and

$$k = \frac{d^2V_{\rm ad}}{dx^2} = \frac{1}{2}\left(\frac{d^2V_1}{dx^2} + \frac{d^2V_2}{dx^2}\right) - \frac{1}{4}C', \tag{S6}$$

where

$$\begin{aligned} C' = \; & -\frac{1}{2}\left[(V_1 - V_2)^2 + 4H_{\rm SO}^2\right]^{-3/2} \\ & \times \left[2(V_1 - V_2)\left(\frac{dV_1}{dx} - \frac{dV_2}{dx}\right) + 8H_{\rm SO}\frac{dH_{\rm SO}}{dx}\right]^2 \\ & + \left[(V_1 - V_2)^2 + 4H_{\rm SO}^2\right]^{-1/2} \\ & \times \Bigg\{2\left[\left(\frac{dV_1}{dx} - \frac{dV_2}{dx}\right)^2 + (V_1 - V_2)\left(\frac{d^2V_1}{dx^2} - \frac{d^2V_2}{dx^2}\right)\right] \\ & \quad + 8\left(\frac{dH_{\rm SO}}{dx}\right)^2 + 8H_{\rm SO}\frac{d^2H_{\rm SO}}{dx^2}\Bigg\}. \end{aligned} \tag{S7}$$

At the MECP, $V_1 - V_2 = 0$ and we also assume that the spin-orbit coupling constant does not vary in the vicinity of the MECP, i.e., its derivatives are zero. Then the final expression for the force constant is:

$$k_{\rm ad} = \frac{1}{2}\left(\frac{d^2V_1}{dx^2} + \frac{d^2V_2}{dx^2}\right) - \frac{1}{4H_{\rm SO}}\left(\frac{dV_1}{dx} - \frac{dV_2}{dx}\right)^2. \tag{S8}$$

or equivalently:

$$k_{\mathrm{ad}} = \frac{1}{2}(k_1 + k_2) - \frac{1}{4H_{\mathrm{SO}}}|\Delta g|^2. \tag{S9}$$

In Eq. S9, k_1 and k_2 are always positive for the case of a peaked intersection of two parabolic potentials and have arbitrary sign otherwise.

We require $k_{\mathrm{ad}} < 0$ for the adiabatic potential to describe the transition barrier in the vicinity of the crossing point. In the present case, this holds true in the regime when the spin-orbit constant is smaller than the critical value:

$$H_{\mathrm{SO}}^{\mathrm{cr}} = \frac{|\Delta g|^2}{2(k_1 + k_2)}. \tag{S10}$$

At $H_{\mathrm{SO}} = H_{\mathrm{SO}}^{\mathrm{cr}}$,$k_{\mathrm{ad}} = 0$, and the barrier disappears (Figure S3).

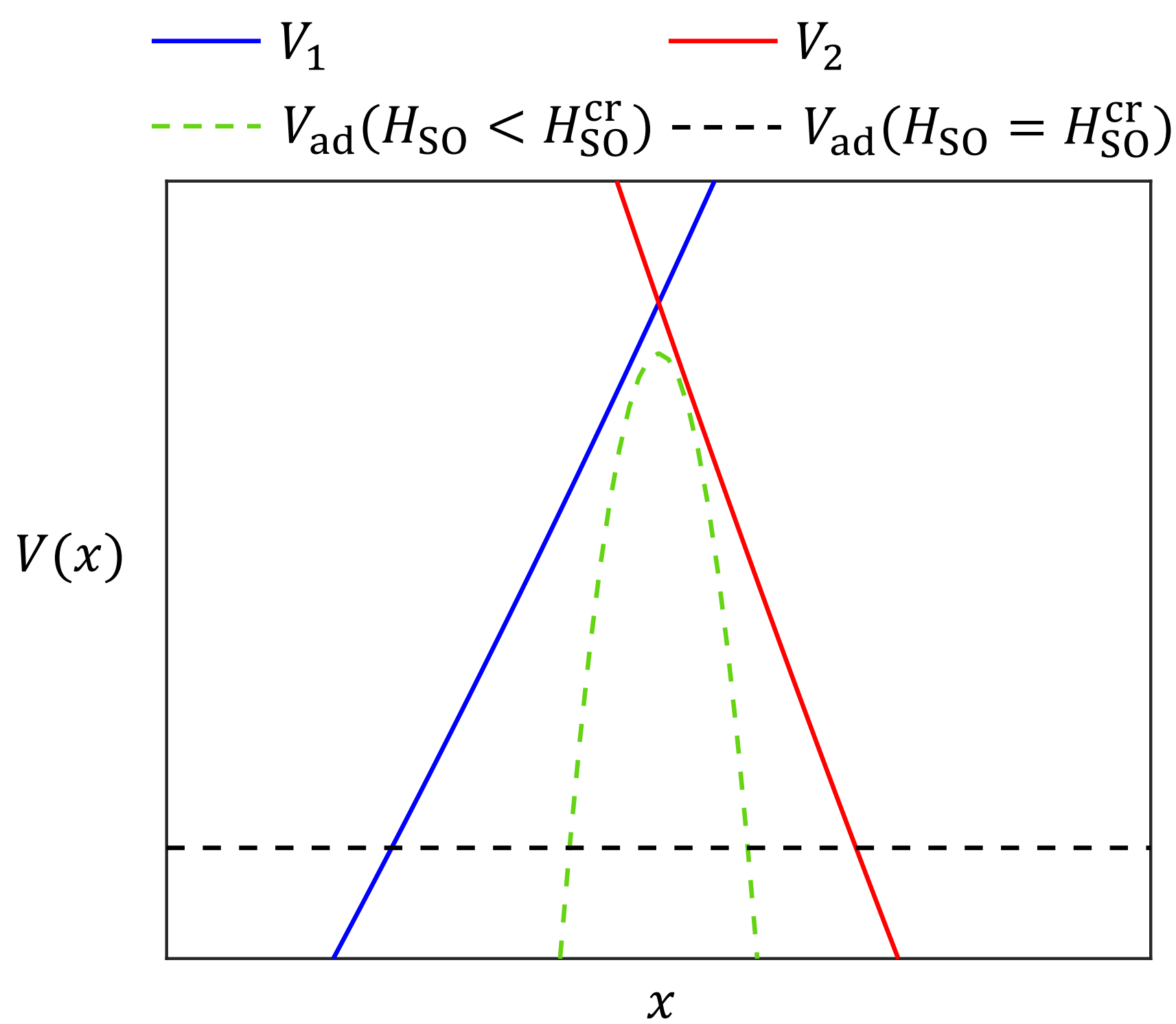

Figure S3: The 1D crossing potentials of two spin-diabatic states (solid lines) and two spin-adiabatic potentials calculated from k_{ad} (dashed lines)

5 Integration step size

Here, we estimate the convergence of the rate constant with respect to the numerical integration step size using examples of the isomerization reaction of propylene oxide to acetone at $T = 1000$ K and the triplet state relaxation in thiophosgene at $T = 300$ K.[2,4] We estimate the step-size sensitivity as:

$$\delta_h = \frac{\left|k\left(\frac{h}{2}\right) - k(h)\right|}{\left|k\left(\frac{h}{2}\right)\right|}, \tag{S11}$$

where h is the integration step size. Figure S4 below shows the sensitivity δ_h calculated at $h =$ 1.0, 0.5, 0.25, and 0.125 cm^{-1} for both examples.

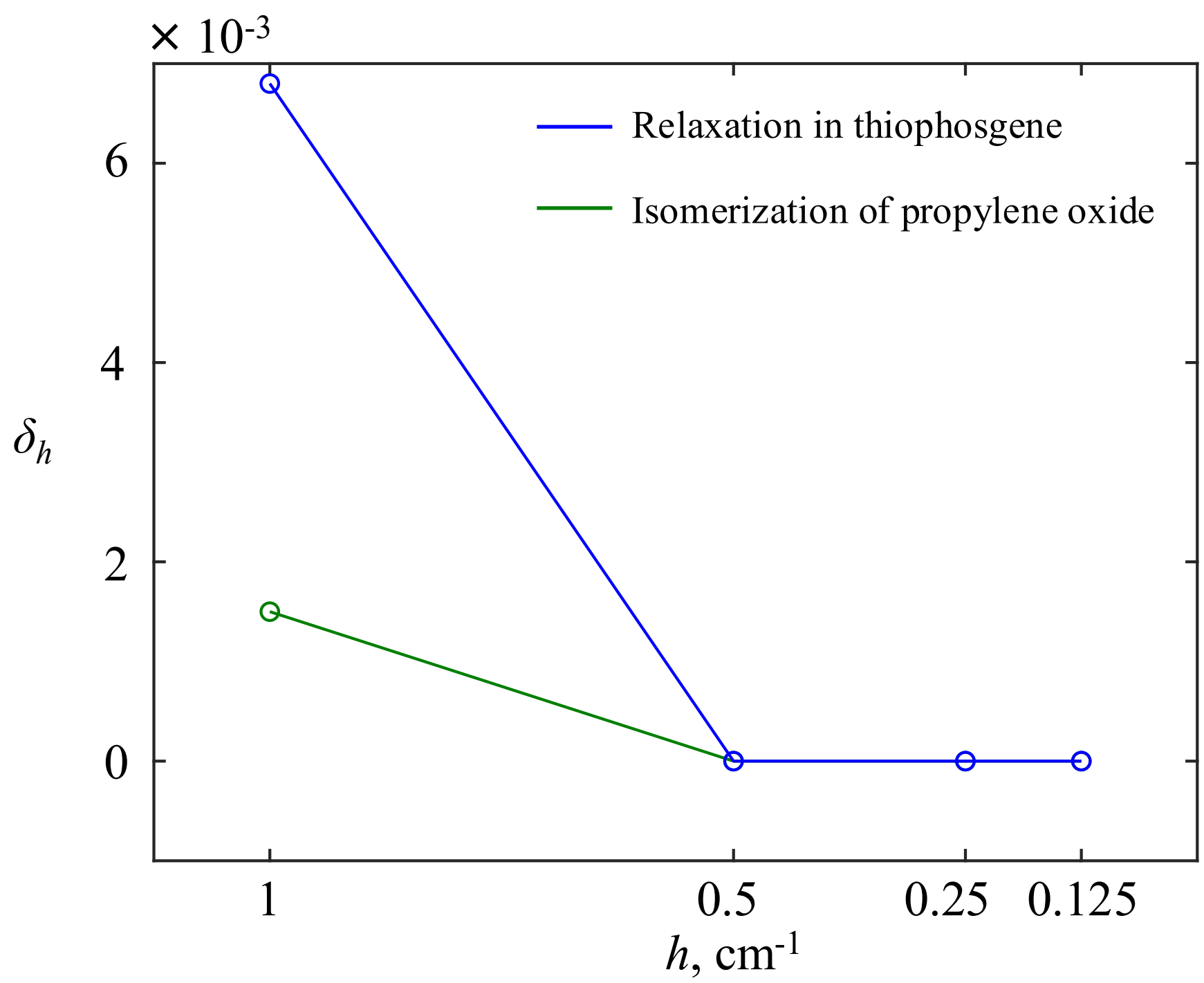

Figure S4: Convergence of the rate constants of the isomerization of propylene oxide to acetone and the triplet state relaxation in thiophosgene with respect to the integration step size h.

At $h = 1$ cm^{-1}, the relaxation rate of thiophosgene converges approximately three times

more slowly than the isomerization rate of propylene oxide, as can be seen from comparing the δ_h values for two molecules. This can be attributed to a more sparse rovibrational density of states in thiophosgene given its smaller size. The convergence analysis indicates that the integration step size of 1.0 cm^{-1} is sufficiently small in both examples.

6 XYZ coordinates

Table S4: Optimized atomic coordinates of TTM-1Cz-An $^2[D_1S_0]$ state.

Atom	X	Y	Z
C	2.670546	-1.384397	0.635301
C	2.076056	-0.096345	0.413891
C	0.897154	0.112261	-0.408695
C	2.591108	1.135025	0.987475
C	0.345054	1.342397	-0.645136
C	2.016210	2.362095	0.791599
C	0.884146	2.501127	-0.042387
H	-0.567103	1.408062	-1.236381
H	2.493476	3.243725	1.216831
C	3.106507	-1.770588	1.971601
C	2.321727	-1.519969	3.127035
C	4.339561	-2.419417	2.244891
C	2.708572	-1.853571	4.416929
C	4.741825	-2.787477	3.519584
C	3.922124	-2.500501	4.606416
H	2.054806	-1.634538	5.259967
H	5.706498	-3.271490	3.665979

Atom	X	Y	Z
C	2.838335	-2.312769	-0.476916
C	3.316264	-1.896197	-1.746132
C	2.538214	-3.698464	-0.403062
C	3.469705	-2.742045	-2.835169
C	2.704833	-4.572844	-1.466233
C	3.170319	-4.089217	-2.684591
H	3.845098	-2.355349	-3.781574
H	2.444088	-5.624062	-1.350740
Cl	0.749721	-0.793145	2.950864
Cl	5.485945	-2.712282	0.968083
Cl	4.422284	-2.950617	6.206565
Cl	1.837276	-4.375750	1.039056
Cl	3.792433	-0.237607	-1.977978
Cl	3.372018	-5.175648	-4.023642
Cl	0.068550	-1.261138	-1.062900
Cl	4.067299	1.092508	1.892952
C	0.061862	4.725037	0.708089
C	-0.109842	4.247658	-1.507604
C	0.242679	4.671618	2.090375
C	-0.523314	5.849856	0.091963
C	-0.003681	3.674576	-2.775655
C	-0.634038	5.544622	-1.326391
C	-0.129996	5.784817	2.835971
H	0.645487	3.788837	2.585698
C	-0.893872	6.950387	0.857718
C	-0.478600	4.409961	-3.857618

Atom	X	Y	Z
H	0.447395	2.694693	-2.928207
C	-1.099452	6.265807	-2.422328
C	-0.689798	6.929047	2.240912
H	0.006344	5.766522	3.919104
H	-1.349919	7.824251	0.387730
C	-1.027567	5.686528	-3.687054
H	-0.411610	3.981764	-4.859653
H	-1.505030	7.271199	-2.292948
H	-1.389590	6.237890	-4.556794
N	0.314357	3.747119	-0.265822
C	-1.079650	8.101566	3.081719
C	-0.109008	9.056126	3.448708
C	-2.416715	8.247927	3.505021
C	1.262084	8.958825	3.037478
C	-0.489256	10.181227	4.258427
C	-2.786903	9.378466	4.311795
C	-3.440246	7.300503	3.168748
C	2.179802	9.898813	3.406840
H	1.567662	8.115583	2.417387
C	0.505487	11.144260	4.624638
C	-1.816249	10.315186	4.668730
C	-4.147922	9.516712	4.735728
H	-3.176688	6.432150	2.564341
C	-4.726324	7.467743	3.593751
C	1.798804	11.010237	4.214870
H	3.217887	9.803577	3.081013

Atom	X	Y	Z
H	0.200543	11.992576	5.241979
H	-2.101803	11.172671	5.283262
C	-5.089746	8.594177	4.388893
H	-4.413263	10.382566	5.347009
H	-5.487992	6.732282	3.325853
H	2.548284	11.751345	4.500494
H	-6.124253	8.710106	4.718883

Table S5: Optimized atomic coordinates of TTM-1Cz-An $^2[D_0T_1]$ state.

Atom	X	Y	Z
C	2.631974	-1.393457	0.638106
C	2.048790	-0.063067	0.400100
C	0.851063	0.119013	-0.324137
C	2.647461	1.122151	0.878747
C	0.289760	1.367487	-0.548851
C	2.110231	2.381005	0.652735
C	0.920876	2.511509	-0.062621
H	-0.657712	1.442321	-1.080778
H	2.639298	3.262500	1.012306
C	3.039098	-1.787410	1.998197
C	2.184823	-1.643887	3.112614
C	4.310126	-2.334406	2.276670
C	2.556260	-2.010564	4.399458
C	4.704444	-2.714377	3.552796
C	3.819667	-2.547641	4.610306

Atom	X	Y	Z
H	1.855238	-1.892843	5.224442
H	5.701680	-3.119514	3.717873
C	2.809021	-2.331780	-0.484100
C	3.437986	-1.954660	-1.690080
C	2.362071	-3.669376	-0.427236
C	3.611712	-2.828616	-2.755168
C	2.528096	-4.565015	-1.475413
C	3.154383	-4.134823	-2.638042
H	4.116977	-2.493399	-3.659746
H	2.153249	-5.583693	-1.387686
Cl	0.558336	-1.065652	2.913921
Cl	5.503526	-2.484802	1.023061
Cl	4.297262	-3.013755	6.209382
Cl	1.485833	-4.252017	0.955170
Cl	4.117806	-0.366439	-1.875937
Cl	3.366827	-5.238963	-3.956505
Cl	-0.056595	-1.250983	-0.890936
Cl	4.178351	1.070428	1.700724
C	0.077952	4.739507	0.685697
C	-0.000712	4.306249	-1.535539
C	0.230821	4.672744	2.071679
C	-0.474320	5.881460	0.068132
C	0.126041	3.743242	-2.807451
C	-0.522794	5.605504	-1.355103
C	-0.158988	5.774588	2.822218
H	0.637299	3.786790	2.560293

Atom	X	Y	Z
C	-0.858829	6.976593	0.844493
C	-0.298669	4.498390	-3.894209
H	0.548638	2.748473	-2.951136
C	-0.943896	6.345549	-2.463147
C	-0.699722	6.933628	2.229146
H	-0.045479	5.743194	3.908093
H	-1.286112	7.863990	0.371999
C	-0.833182	5.785320	-3.728935
H	-0.210572	4.078572	-4.898649
H	-1.349978	7.351061	-2.333086
H	-1.158471	6.350371	-4.604567
N	0.362850	3.785212	-0.291836
C	-1.098109	8.092030	3.074771
C	-0.123829	9.121305	3.382378
C	-2.453882	8.175306	3.582821
C	1.186650	9.072618	2.910964
C	-0.514079	10.235455	4.200479
C	-2.830002	9.295769	4.399027
C	-3.414124	7.203260	3.309041
C	2.118935	10.085327	3.222441
H	1.499237	8.234575	2.286991
C	0.421789	11.225274	4.496252
C	-1.856242	10.302439	4.691934
C	-4.133188	9.376963	4.887092
H	-3.145475	6.345551	2.691321
C	-4.728333	7.304372	3.813455

Atom	X	Y	Z
C	1.741341	11.153130	4.008339
H	3.137581	10.014463	2.835558
H	0.117662	12.069898	5.119171
H	-2.147805	11.152146	5.313230
C	-5.086446	8.381499	4.595707
H	-4.412288	10.232314	5.507126
H	-5.454866	6.523905	3.578175
H	2.455107	11.941820	4.255079
H	-6.100678	8.469009	4.990742

Table S6: Optimized atomic coordinates of $[Fe^{II}(bqn)(phen)(Ph)Cl]^0$ complex reactant quintet state.

Atom	X	Y	Z
C	-1.139095	-0.398524	-4.327960
C	0.073003	-0.458799	-4.994691
C	1.276283	-0.288544	-4.270252
C	2.580640	-0.335502	-4.873700
C	3.709905	-0.164091	-4.125098
C	3.632823	0.073150	-2.708667
C	4.770036	0.259398	-1.888980
C	4.601687	0.479864	-0.532910
C	3.295547	0.515944	-0.004166
N	2.210457	0.353226	-0.749463
C	2.359041	0.129295	-2.077470
C	1.164470	-0.060514	-2.872111
N	0.364857	2.112474	1.038087

Atom	X	Y	Z
C	-1.148793	-0.175408	-2.936386
H	-2.084629	-0.526466	-4.858985
H	0.113541	-0.636782	-6.073047
H	2.651289	-0.514954	-5.949887
H	4.697034	-0.205261	-4.593197
H	5.768515	0.223710	-2.333626
H	5.457222	0.622540	0.130370
H	3.134754	0.677433	1.065522
H	-2.073348	-0.116005	-2.350229
C	0.967604	-0.684375	4.210478
C	1.910433	-0.384422	5.214407
C	1.371434	1.476692	3.163037
C	0.041788	3.044291	0.147518
C	1.134790	2.449383	2.106339
C	2.551531	0.843806	5.219073
C	2.290732	1.801788	4.209007
C	0.518108	4.366864	0.206323
C	1.711731	3.751865	2.216395
C	2.904404	3.102359	4.238519
C	1.380307	4.709575	1.231428
C	2.607385	4.046813	3.300904
C	0.696677	0.234552	3.208662
H	-0.073995	0.009610	2.470651
H	2.121684	-1.115138	5.999713
H	-0.643022	2.725601	-0.644704
H	3.261439	1.094959	6.012282

Atom	X	Y	Z
H	0.217517	5.088754	-0.555863
H	3.599190	3.330254	5.051815
H	1.802748	5.716476	1.299822
H	3.052181	5.044546	3.351709
Fe	0.016958	-0.012975	-0.021932
Cl	-2.283086	0.546358	0.117526
H	2.501826	-2.195015	0.346788
C	1.560992	-2.707238	0.583092
C	0.337882	-2.006073	0.517549
C	-0.820503	-2.753972	0.838154
H	-1.798540	-2.260939	0.801380
C	-0.761536	-4.105061	1.204554
H	-1.682603	-4.646781	1.445547
C	0.471173	-4.764535	1.262384
H	0.522872	-5.819871	1.547990
C	1.636752	-4.058266	0.948933
H	2.608879	-4.561946	0.989845
H	0.440271	-1.641021	4.215805
N	-0.029957	-0.015337	-2.237910

Table S7: Optimized atomic coordinates of $[Fe^{II}(bqn)(phen)(Ph)Cl]^0$ complex quintet transition state.

Atom	X	Y	Z
C	2.087685	1.052480	-4.364191
C	0.733321	1.215092	-4.602922
C	-0.187525	0.981324	-3.555844

Atom	X	Y	Z
C	-1.611459	1.113687	-3.707720
C	-2.458204	0.867365	-2.664983
C	-1.953811	0.474926	-1.376764
C	-2.783398	0.207734	-0.263798
C	-2.205605	-0.161462	0.939315
C	-0.802265	-0.255293	1.025187
N	0.001282	-0.007798	-0.002816
C	-0.549672	0.343754	-1.189073
C	0.343210	0.591029	-2.296409
N	2.074462	-2.079122	0.606753
C	2.519492	0.654390	-3.084375
H	2.826665	1.225903	-5.149176
H	0.367066	1.520861	-5.586977
H	-2.005260	1.415272	-4.682042
H	-3.539347	0.967930	-2.792748
H	-3.868781	0.296325	-0.363648
H	-2.814872	-0.374831	1.820015
H	-0.317331	-0.530631	1.966246
H	3.576732	0.502890	-2.841074
C	2.480074	0.897909	3.660635
C	2.593069	0.489305	5.005276
C	2.311868	-1.391660	2.926163
C	1.976897	-3.005799	-0.339426
C	2.183584	-2.433307	1.913411
C	2.564209	-0.856970	5.317754
C	2.425980	-1.821295	4.291968

Atom	X	Y	Z
C	1.967107	-4.381691	-0.063333
C	2.175990	-3.815445	2.281984
C	2.406781	-3.221018	4.622087
C	2.063905	-4.781098	1.258117
C	2.287418	-4.182315	3.664171
C	2.335187	0.000389	2.603553
H	2.281953	0.965417	1.518846
H	2.705036	1.236116	5.797306
H	1.916983	-2.643265	-1.370087
H	2.651888	-1.194852	6.354376
H	1.887996	-5.103901	-0.878327
H	2.496643	-3.502522	5.675488
H	2.061113	-5.842056	1.524871
H	2.279744	-5.243389	3.927987
Fe	2.325824	-0.009182	-0.003911
Cl	4.527213	-0.386824	-0.657594
H	0.137205	2.483040	1.241352
C	1.071412	2.955867	0.916511
C	2.219600	2.165105	0.713185
C	3.402909	2.841087	0.345441
H	4.320434	2.263675	0.193149
C	3.426034	4.228100	0.152741
H	4.357446	4.722517	-0.141655
C	2.264718	4.984451	0.343595
H	2.281491	6.068887	0.198478
C	1.083771	4.343750	0.734651

Atom	X	Y	Z
H	0.173178	4.928521	0.901835
H	2.508530	1.971115	3.439351
N	1.672296	0.430204	-2.082698

Table S8: Optimized atomic coordinates of [Fe^{II}(bqn)(phen)(Ph)Cl]^0 complex product quintet state.

Atom	X	Y	Z
C	0.407322	1.084359	-4.419038
C	1.770658	1.232162	-4.616422
C	2.654939	1.006582	-3.536187
C	4.085691	1.111084	-3.640654
C	4.891796	0.858145	-2.567105
C	4.338133	0.472742	-1.296607
C	5.122891	0.171824	-0.159556
C	4.495906	-0.207483	1.015441
C	3.088782	-0.280512	1.051383
N	2.327016	0.000768	0.000353
C	2.926931	0.371275	-1.156166
C	2.075473	0.648847	-2.288302
N	-0.175338	2.122103	0.594683
C	-0.070297	0.726437	-3.142376
H	-0.302522	1.236883	-5.234815
H	2.171185	1.510939	-5.595181
H	4.518748	1.394467	-4.603767
H	5.978375	0.937050	-2.659329
H	6.212841	0.237754	-0.220087

Atom	X	Y	Z
H	5.070514	-0.449624	1.911929
H	2.558424	-0.572606	1.962690
H	-1.134211	0.564970	-2.938682
C	-0.032600	-1.348709	2.938761
C	-0.140406	-1.250504	4.344721
C	-0.189401	1.033045	2.751389
C	-0.252036	3.207197	-0.167367
C	-0.253992	2.241578	1.950852
C	-0.265514	-0.012877	4.957949
C	-0.297641	1.162395	4.171623
C	-0.409131	4.502296	0.354959
C	-0.408221	3.520158	2.567410
C	-0.442143	2.466379	4.762619
C	-0.483416	4.654434	1.730441
C	-0.493506	3.597808	3.999860
C	-0.043456	-0.229452	2.100844
H	-0.126930	-2.158787	4.956243
H	-0.188856	3.051100	-1.248892
H	-0.347686	0.067661	6.046023
H	-0.470469	5.358725	-0.319820
H	-0.517411	2.537360	5.852237
H	-0.605110	5.645310	2.178284
H	-0.609275	4.581531	4.463522
Fe	0.000178	-0.000018	0.000038
Cl	-1.868347	-0.933166	-0.950696
H	0.056646	-2.351920	2.504127

Atom	X	Y	Z
N	0.739742	0.529991	-2.105862

Table S9: Optimized atomic coordinates of benzene singlet state.

Atom	X	Y	Z
H	0.027204	0.551542	2.761650
C	1.082628	0.265355	2.756021
C	1.760144	0.110238	1.541691
C	3.110604	-0.256039	1.535538
C	3.783528	-0.467163	2.744101
H	4.839003	-0.753359	2.739070
C	3.106184	-0.312067	3.958423
H	3.632259	-0.477070	4.902857
C	1.755949	0.054138	3.964409
H	1.226108	0.175452	4.913374
H	1.234241	0.275187	0.597080
H	3.640310	-0.377349	0.586560

References

(1) Neese, F. Software update: the ORCA program system, version 6.0. *WIRES Comput. Molec. Sci.* **2025**, *15*, e70019.

(2) Dergachev, V. D.; Rooein, M.; Dergachev, I. D.; Lykhin, A. O.; Mauban, R. C.; Varganov, S. A. NAST: Nonadiabatic Statistical Theory Package for Predicting Kinetics of Spin-Dependent Processes. *Topics in Current Chemistry* **2022**, *380*, 1–15.

(3) Gorgon, S. et al. Reversible spin-optical interface in luminescent organic radicals. *Nature* **2023**, *620*, 538–544.

(4) Lykhin, A. O.; Varganov, S. A. Intersystem crossing in tunneling regime: T1 → S0 relaxation in thiophosgene. *Phys. Chem. Chem. Phys.* **2020**, *22*, 5500–5508.
